# Detailed Structural Elucidation of Antibody-Drug Conjugate Biotransformation Species Using High Resolution Multiple Reaction Monitoring Mass Spectrometry with Orthogonal Dissociation Methods

**DOI:** 10.1021/acsptsci.4c00445

**Published:** 2024-12-17

**Authors:** Junyan Yang, Hui Yin Tan, Jiaqi Yuan, Yue Huang, Anton I. Rosenbaum

**Affiliations:** Integrated Bioanalysis, Clinical Pharmacology and Safety Sciences, R&D, AstraZeneca, 121 Oyster Point Blvd, South San Francisco, California 94080, United States

**Keywords:** AZD8205, puxitatug samrotecan, high-resolution mass spectrometry, multiple reaction monitoring, collision-induced dissociation, electron-activated dissociation

## Abstract

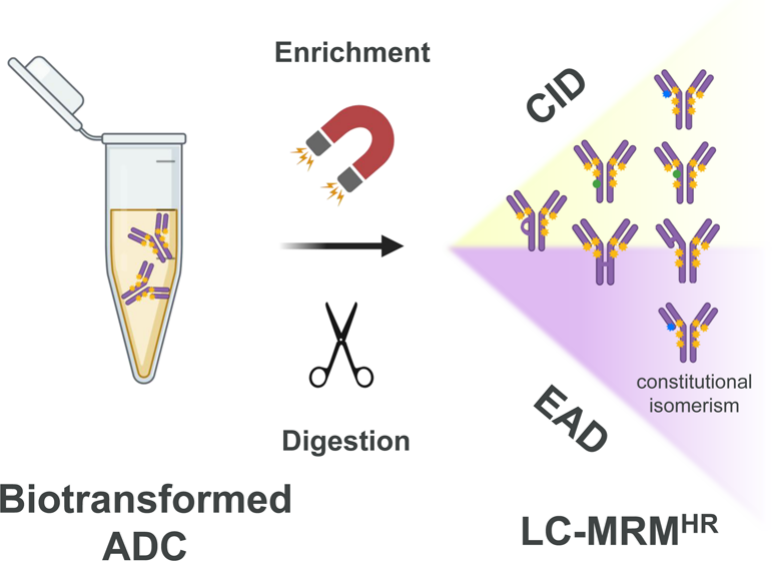

Antibody-drug conjugates (ADCs) are a promising drug modality substantially expanding in both the discovery space and clinical development. Assessing the biotransformation of ADCs *in vitro* and *in vivo* is important in understanding their stability and pharmacokinetic properties. We previously reported biotransformation pathways for the anti-B7H4 topoisomerase I inhibitor ADC, AZD8205, puxitatug samrotecan, that underpin its structural stability *in vivo* using an intact protein liquid chromatography-high resolution mass spectrometry (LC-HRMS) approach. Herein, we employed a LC-high resolution multiple reaction monitoring (LC-MRM^HR^) approach using both collision-induced dissociation (CID) and electron-activated dissociation (EAD) methods, confirming our earlier findings. Furthermore, we were able to obtain additional detailed structural information on the biotransformation products expanding on earlier intact analyses. We also highlight the high sensitivity of LC-MRM^HR^ for successfully identifying minor biotransformation products at low concentrations that were not detectable using the intact protein LC-HRMS workflow. Especially, EAD aided in the confirmation of biotransformation species that contain newly formed disulfide bonds due to the preferential dissociation of disulfide bonds using this method. We observed biotransformation reactions that vary between linker-payload (PL) conjugation sites on the antibody. For example, the trend toward constitutional isomerism in thio-succinimide linker hydrolysis, and the resulting positional isomers from thiol adduct formation following linker-PL deconjugation. The reported orthogonal analytical approaches highly complement and fortify the intact protein LC-HRMS data. This study sheds further light on detailed structural characterization of various ADC species and validates the proposed biotransformation pathways explaining the stability of AZD8205 *in vivo*.

Antibody-drug conjugates (ADCs) are a fast-growing biotherapeutic modality that precisely delivers cytotoxic payload (PL) to targeted cells.^1,2^ Recently, ADCs have shown great promise in oncology,^3,4^ and there is an increasing interest to expand the development of ADCs with diverse mechanisms of action and other therapeutic areas.^5^ ADC is composed of three parts, antibody backbone, linker, and PL. The antibody binds to target molecules and directs the delivery of the PL. In oncology setting, the PL typically targets one of the two cellular functions, microtubule-dependent mitosis or DNA replication, to induce cell apoptosis.^1,6,7^ Due to the acute toxicity of the PL, ADCs are designed to remain intact during circulation, to avoid off-target toxicity.^8^ However, *in vitro* or *in vivo* biotransformation of ADCs may occur because of the presence of enzymes^9^ or variations in redox potentials.^10^ Most importantly, each individual ADC may undergo distinctive biotransformation pathways, largely defined by the linker design, selection of conjugation sites and methods, and PL properties.^11−15^ Thus, it is essential to characterize the biotransformation products of ADCs in detail to understand their stability and pharmacokinetic (PK) properties.^16^

In the past few years, researchers investigated ADC biotransformation from various perspectives.^17,18^ The biotransformation of ADCs could stem from linker hydrolysis,^14,19,20^ antibody modification,^21^ linker-PL deconjugation,^12^ and PL metabolism.^12,22^ Previously, we reported an unexpected deamidation reaction occurring in the complementarity-determining regions of the antibody backbone of an ADC, MEDI7247.^23^ The change from asparagine to isoaspartic acid leads to decreased binding affinity between the ADC and its target, thus potentially compromising its overall efficacy.^24^ Su et al. reported the linker-PL deconjugation from an anti-HER2 THIOMAB ADC.^12^ The premature release of PL could result in decreased efficacy and undesired cytotoxicity.^25^ They also reported that the tubulysin PL from a THIOMAB ADC went through a deacetylation reaction, leading to inactive PL.^12^ Such a biotransformation could compromise its efficacy.

The biotransformation of ADCs may not only impact their pharmacological profiles but also may pose challenges for their quantification in study samples.^14,23^ One common workflow for determining the concentrations of total antibody and ADCs is using a ligand binding assay (LBA).^26,27^ In this context, understanding the biotransformation of ADCs provides insight into appropriate selection of capture and detection reagents that should not be affected by a given biotransformation for enrichment and subsequent quantitative analysis.^23,28^ Another widely used approach is monitoring the surrogate analyte to represent ADC concentration through LC-MRM approach.^29,30^ In many established quantitative intact ADC assays, the PL itself serves as a surrogate analyte after enzymatic or chemical release from the antibody.^31,32^ However, most potential biotransformation on PL would result in mass to charge (*m*/*z*) and possibly retention time (RT) mismatch with the prespecified values. Both cases could lead to the inaccurate quantification of ADC, compromising the correct characterization of its PK profile.

A common approach to characterize ADC biotransformation utilizes hybrid LBA capture coupled with intact protein mass liquid chromatography-high-resolution mass spectrometry (LBA-LC-HRMS).^12,33^ First, the ADC is selectively enriched from samples through immunocapture, then eluted off the substrate before being analyzed through LC-HRMS.^34^ Finally, data analysis and structure elucidation rely on protein deconvolution by integrating information from RT—*m*/*z* domain to RT—deconvoluted mass domain.^35,36^ Through comparing the changes in deconvoluted mass, scientists may identify and propose the potential structures of biotransformation species.^37^ Previously, our group employed this method to understand the biotransformation profiles of multiple ADCs with different designs and therapeutic purposes.^14,23,31,35^ For AZD8205, a B7H4-directed ADC, with drug to antibody ratio (DAR) equal to 8,^38^ we observed several important biotransformation phenomena, including the progression of thio-succinimide linker hydrolysis and linker-PL deconjugation *in vivo*. Interestingly, we also found a product that represents the disulfide bond reformation between antibody light chain (Lc) and heavy chain (Hc), as a cysteine-conjugated DAR 8 ADC should not contain interchain disulfide bonds. Although the intact protein LBA-LC-HRMS method is robust and routinely used for the characterization of ADC biotransformation, the structural information still heavily relies on the deconvoluted mass changes. Further structural confirmation and characterization can improve confidence in the findings reported.

In this work we developed a method that is suitable for the in-depth structural elucidation of ADC biotransformation species. Immunoaffinity capture allows selective isolation of ADCs from matrices, while on-beads tryptic digestion yields peptides containing possible biotransformation modifications ready for assessment by LC-MRM^HR^. The bottom-up method avoids the possible limitation where biotransformation peaks are overwhelmed by parent/untransformed ADC peak in intact protein LBA-LC-HRMS. Furthermore, MS/MS dissociation enabled detailed structural confirmation of biotransformation products through mass fingerprinting.^39^ Many of the findings in this work support our earlier results.^35^ Traditional LBA-LC-MRM identifies the surrogate analyte by filtering precursor ions in Q1 and then detecting qualified product ions in Q3. Although effective, the sensitivity could be low because the low-resolution nature of triple quadrupole instruments leads to the increased noise level.^40^ Thus, LBA-LC-MRM^HR^ is more suitable for the specific characterization of low concentration level biotransformation products in a complex matrix.

Collision-induced dissociation (CID), a well-established dissociation method, has been routinely used for the characterization of proteins, peptides, and small molecules, especially in quantitative assays.^41^ In general, it dissociates the weakest bonds of precursor ions, generating *b*/*y* ions for peptides.^42^ Compared to CID, electron-based dissociation (ExD) is often considered as a gentler dissociation method by transferring or attaching electrons to precursor ions.^43^ It predominantly dissociates the peptide backbone, producing *c*/*z* ions and preserving the more labile side chain modifications, making itself ideal for identifying post-translational modifications.^44^ Besides, ExD showcased the preferential cleavage of disulfide bonds, providing important information in pinpointing disulfide linkage sites.^45^ In this study, CID could inform specific localization of linker-PL deconjugation and subsequent positional isomerism in forming the thiol adduct, whereas intact LBA-LC-HRMS was unable to discern site-specific changes. We also highlight the advantages of the newly developed electron-activated dissociation (EAD). Previously, scientists employed EAD to investigate the glucuronidation and oxidation metabolism of a small-molecule therapeutic.^46^ In our case, EAD not only aided in distinguishing the isomerism of thio-succinimide hydrolysis but also provided supporting data on disulfide bond-linked biotransformation species due to its preferential dissociation of disulfide bonds.^47^ Herein, we present the first such detailed structural characterization of biotransformation products of an ADC from *in vitro* samples using a LBA-LC-MRM^HR^ approach. The findings support the prior conclusion that AZD8205 is structurally stable, with very minor fraction going through unique biotransformation reactions requiring exquisite instrumentation and advanced analytical workflows for detection and characterization.^35^

## Materials and Methods

**Figure 1:**
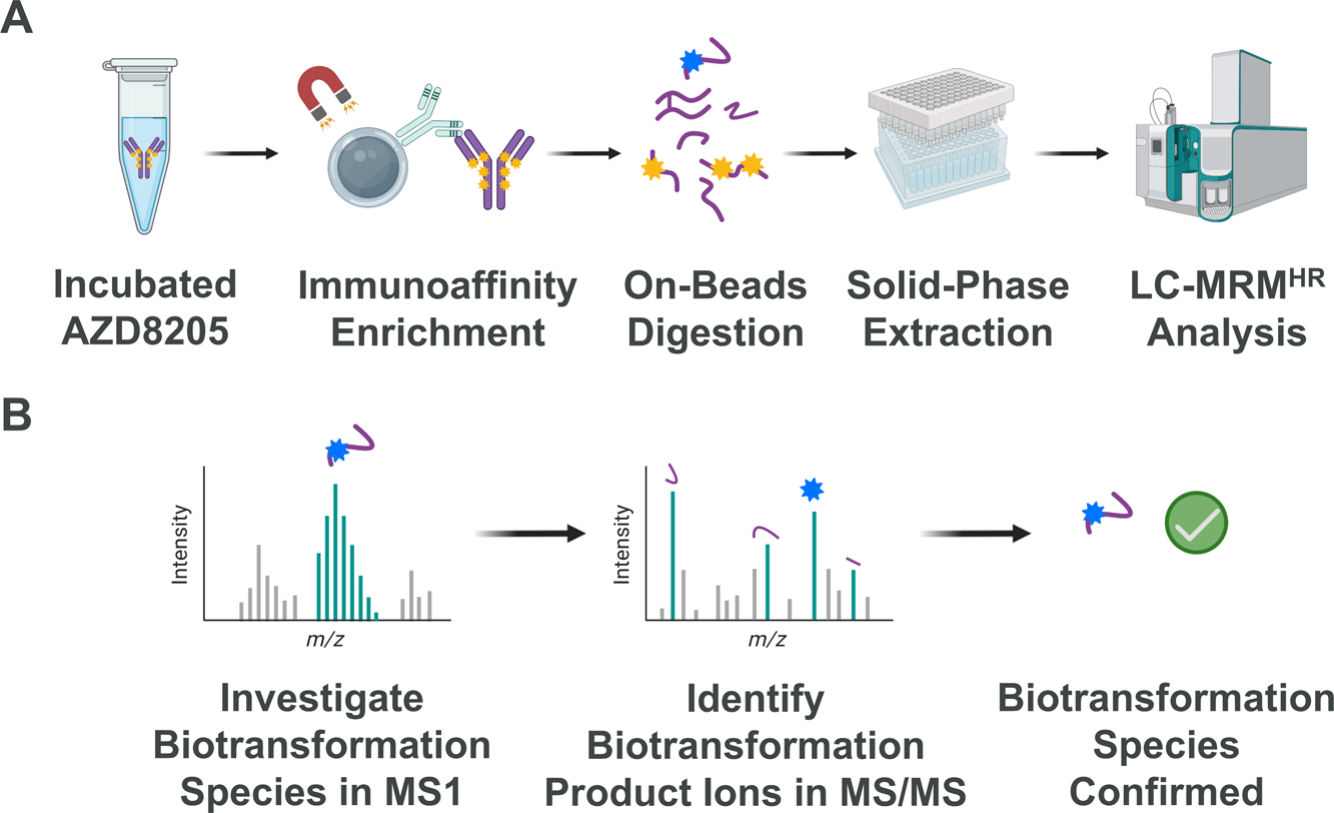
(A) Experimental procedure for preparation of AZD8205 *in vitro* samples to understand its biotransformation products. The workflow includes plasma incubation with AZD8205, immunoaffinity enrichment, trypsin digestion, sample concentration via solid-phase extraction, and finally LC-MRM^HR^ analysis. No reducing agent was used in order to preserve the possible disulfide bonds formed. (B) Data analysis workflow employs a two-step mass fingerprinting to identify the biotransformation species in MS spectrum and elucidate its structure in MS/MS spectrum. Created with BioRender.com.

The materials and methods were reported previously.^48^Figure 1 shows the workflow of experimental steps for sample preparation and LC-MS/MS data analysis. Briefly, 0.1 mg/mL AZD8205 (puxitatug samrotecan, AstraZeneca, Gaithersburg, MD) was incubated for different lengths of time (0, 6, 24, 72, and 168 h) in human or CD1 mouse plasma (BioIVT, Hicksville, NY). Samples were then frozen at -80 °C until analysis. Biotinylated anti-idiotype (anti-ID) antibodies were immobilized via streptavidin on SMART IA beads (ThermoFisher Scientific, Waltham, MA). AZD8205 was enriched using anti-ID conjugated magnetic beads and then digested at 70 °C after activation of the pre-immobilized trypsin on SMART IA beads. The solution containing tryptic digestion products was cleaned-up and concentrated using an Oasis HLB μElution plate (Waters Corporation, Milford, MA) following the manufacturer’s protocol before being injected into Exion LC coupled with SCIEX 7600 ZenoTOF for LC-MS/MS analysis. Digested products were separated through a 74 min LC gradient using a Waters Acquity BEH C18 column (1.7 μm, 2.1 × 50 mm, Waters Corporation, Milford, MA). Although the long LC gradient compromises throughput, we believe it was essential in separating biotransformation products with minor differences, for instance, thio-succinimide hydrolysis constitutional isomers and thiol (Cys or GSH) adduct positional isomers. The structural elucidation of low-concentration biotransformed products also benefited from the extended LC run time to allow the accurate execution of scheduled MRM^HR^. To briefly summarize the LC-MS/MS conditions, the ZenoToF 7600 was operated in a full scan mode, with collision energy (CE) set as 10 V in MS. For CID MRM^HR^, CE = 40 V was used to give sufficient dissociation of peptides in MS/MS. For EAD MRM^HR^, the electron kinetic energy was set as 11 eV. The scheduled MRM^HR^ was performed with specified RT and targeted *m*/*z* values. These parameters were first obtained and tested using an information-dependent acquisition mode.

To identify and analyze the AZD8205 biotransformation products, we employed the LC-MRM^HR^ approach while applying two orthogonal dissociation methods, CID and EAD. The successful confirmation of biotransformation uses a two-step mass fingerprinting approach. First, the monoisotopic *m*/*z* of the biotransformed species in the MS^1^ spectrum was matched. Second, the product ions in the MS/MS spectrum using biotransformed product as precursor ion were also matched. Relative quantification was achieved using the area under the curve (AUC) from MS^1^ XICs, integrated using the MQ4 algorithm in MultiQuant (SCIEX, version 3.0.3863).

## Results and Discussion

This article describes a broadly applicable approach for in-depth investigation of ADC biotransformation products from *in vitro* or *in vivo* studies. The process includes specific enrichment of ADC from samples through immunoaffinity capture and on-bead tryptic digestion, followed by analysis using LC-MRM^HR^. The employment of two orthogonal dissociation methods, CID and EAD, provided complementary results in the characterization of biotransformation species from AZD8205. This method could enable the examination of ADC pharmacological profiles in different studies.

**Figure 2:**
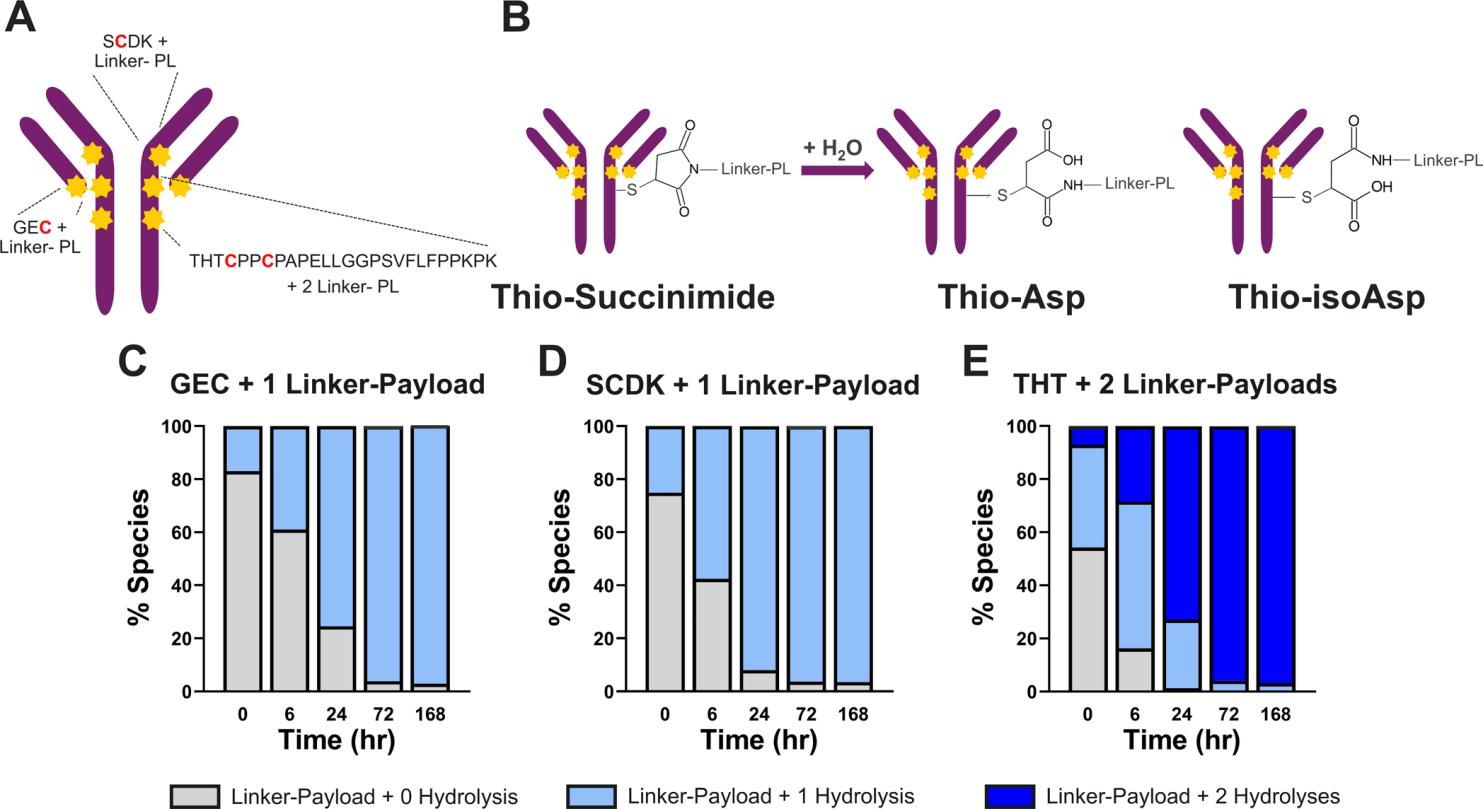
(A) Structure of AZD8205 ADC. Tryptic digestion yields three peptides that contain linker-PL. The red bolded Cs are linker-PL-conjugated cysteines. (B) Thio-succinimide linker hydrolysis reaction. The ring-opening reaction generates two constitutional isomers, thio-aspartic acid (thio-Asp) and thio-isoaspartic acid (thio-isoAsp). The trend of thio-succinimide hydrolysis is shown in (C) GEC-linker-PL, (D) SCDK-linker-PL, and (E) THT-linker-PL. Both GEC and SCDK peptides have one cysteine each that is conjugated to one linker-PL, whereas THT has two cysteines that are conjugated to linker-PLs. The proportion of the various colors within the bars (gray, light blue, and dark blue) in each panel represents the relative abundances of the tryptic peptide with different numbers of hydrolyzed thio-succinimide in linker-PL across different time points. The relative abundances are calculated using area under the curve (AUC) of each species in extracted ion chromatogram (XIC) of MS^1^. XICs were extracted using their theoretical *m*/*z* (Table S1) and a 10 ppm window.

AZD8205, puxitatug samrotecan, is an anti-B7H4 Topoisomerase I inhibitor ADC with an average DAR of 8.^38^ The linker-PL contains maleimide ring, polyethylene glycol (PEG), Val–Ala dipeptide cleavable linker, and AZ14170132 PL. The linker-PL was conjugated to the anti-B7H4 antibody through reduction of all four interchain disulfide bonds, followed by thiol–maleimide reaction with the cysteine side chains’ thiol groups. Trypsin digestion of captured AZD8205 yields three peptides linked with linker-PL: GE**C** from antibody light chain (Lc), S**C**DK from antibody heavy chain (Hc), and THT**C**PP**C**PAPELLGGPSVFLFPPKPK (THT peptide) from the hinge region in Hc, as shown in Figure 2A. All four cysteines in the peptide sequences referenced above were conjugated to linker-PLs.

Previously, we reported AZD8205 major biotransformation species such as thio-succinimide linker hydrolysis and minor linker-PL deconjugation followed by thiol group adduct and disulfide bond formation through intact LC-HRMS.^35^ These previously reported biotransformation pathways demonstrated the structure–stability determinants of AZD8205 conjugation. Herein we employed bottom-up LC-MS/MS using two different dissociation methods to confirm previously observed biotransformation products and reveal further structural details of the AZD8205 biotransformation species.

### Characterization of Thio-Succinimide Hydrolysis Isomerism

The thio-succinimide structures resulting from the thiol–maleimide reaction connect the antibody backbone and linker-PLs. The thio-succinimide ring has been reported to undergo ring-opening hydrolysis.^19^ Previously, we confirmed this major biotransformation pathway on both Lc and Hc by using intact mass LC-HRMS.^35^ Here, we also monitor the linker-PL hydrolysis through a bottom-up LC-MRM^HR^ approach. Figure 2C–E show the percentage ratio between nonhydrolyzed and hydrolyzed thio-succinimide for GEC from Lc, SCDK, and THT from Hc, respectively. These data suggest that over 95% of thio-succinimide was hydrolyzed across all three tryptic products after 72 h incubation in plasma. This finding aligns with our reported observation on the same ADC using an intact LBA-LC-HRMS approach.^35^ Both GEC and SCDK tryptic products show one hydrolysis product, whereas THT shows two hydrolysis products due to two conjugated linker-PLs.

**Figure 3:**
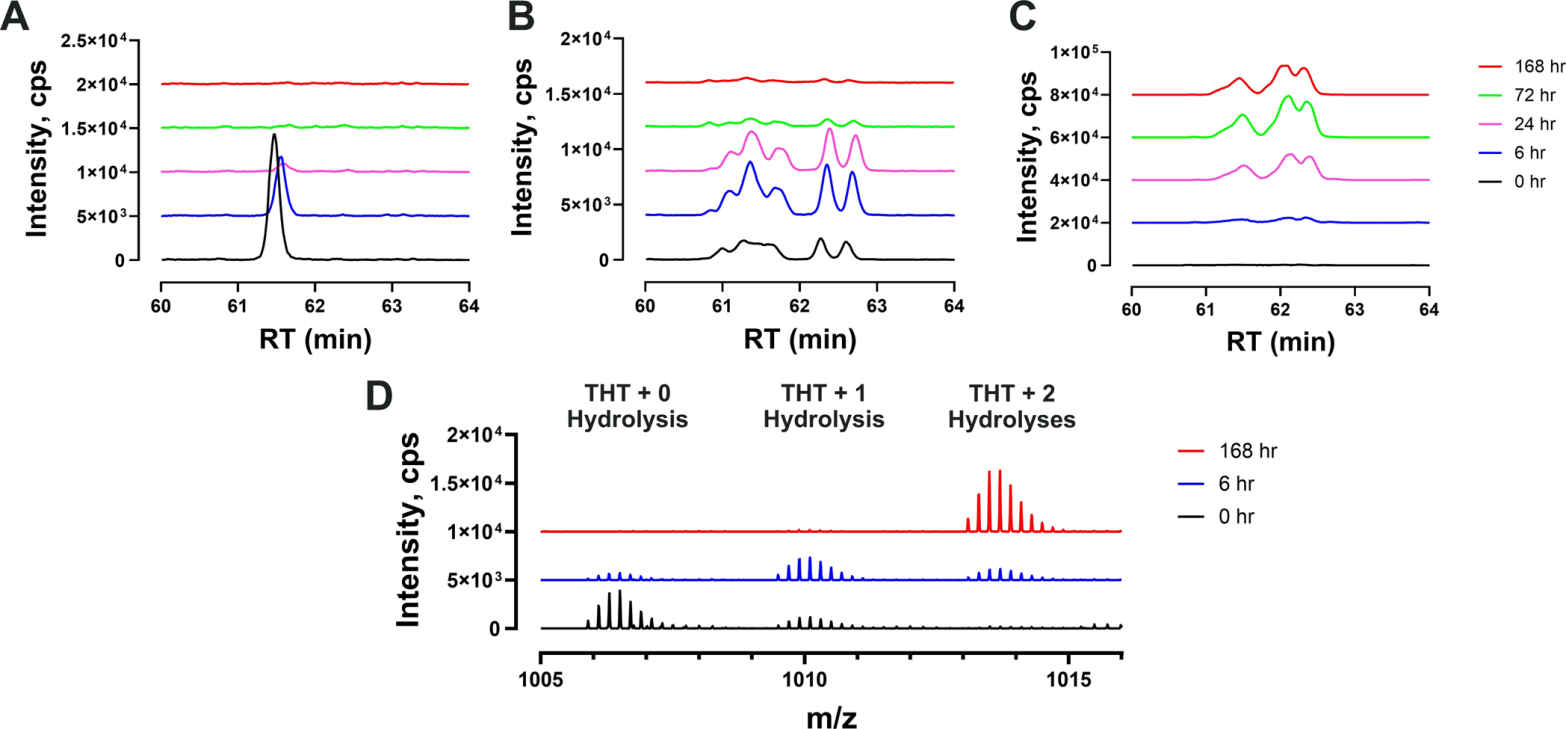
Trend of thio-succinimide hydrolysis in THT-linker-PL at different time points. XICs of THT tryptic peptide from MS^1^ with (A) linker-PL + 0 hydrolysis, (B) linker-PL + 1 hydrolysis, and (C) linker-PLs + 2 hydrolyses. (D) MS^1^ spectra of THT tryptic peptide with different numbers of hydrolyzed linker-PL at selected time points (0, 6, and 168 h). The peaks with monoisotopic *m*/*z* of 1005.9023, 1009.5044, and 1013.1065 are THT with 0, 1, or 2 hydrolyzed linker-PLs (*z* = 5).

Figure 3A–C shows the XICs of non-hydrolyzed, 1 and 2 hydrolyzed thio-succinimide(s) on THT peptide, respectively. The species profile over time suggests greater extent of hydrolysis occurred with longer incubation. No obvious non-hydrolyzed product is observed starting from 72 h of incubation. However, the concentration of species containing one hydrolysis on THT increased at first, then decreased. The concentration of species containing two hydrolysis products increases over time. This suggests both linker-PLs attached to THT peptide are subject to hydrolysis simultaneously. Multiple peaks observed in Figure 3B,C were further investigated and discussed in a later section. Figure 3D shows the representative mass spectra of THT + 0, 1, and 2 hydrolysis products over 0, 6, and 168 h of incubation. The mass spectra confirmed the hydrolysis trends.

Furthermore, we investigated the isomerism of the thio-succinimide hydrolysis products. We successfully assigned structures to each isomeric product through an EAD MS/MS without requiring reference materials using a previously reported approach.^48^ Using the diagnostic ions [R_1_ + Thio + 57 + H]^+^, [R_2_ + Succ + H_2_O-57 + H]^+^, and [R_2_ + ThioSucc + H_2_O-44 + 2H]^2+^, for identifying thio-isoaspartic acid (thio-isoAsp) and [R_2_ + Succ + H_2_O-44 + 2H]^2+^ for identifying thio-aspartic acid (thio-Asp), we confidently assigned the structures to different isomer structures. Figure S1A,B shows the relative abundances of thio-Asp and thio-isoAsp for hydrolyzed linker-PL-containing tryptic peptides, GEC and SCDK, respectively. We observed an ∼3:1 ratio between thio-isoAsp and thio-Asp from SCDK + 1 hydrolyzed linker-PL, as shown in Figure S1B. However, Figure S1A suggests a 1:1 ratio between two isomeric products on the GEC + 1 hydrolyzed linker-PL structure. Geiger and Clarke reported a 3:1 ratio between iso-aspartic acid and aspartic acid resulting from asparagine deamidation.^49^ Since the thio-succinimide hydrolysis mechanism is structurally similar to asparagine deamidation,^50^ one could expect the ratio between two isomers from thio-succinimide hydrolysis might approach 3:1 as well. However, we observed different isomer ratios between linker-PL conjugation sites on GEC and SCDK peptides. Thus, we hypothesize that the thio-succinimide hydrolysis isomerization might be influenced by the location of conjugation sites. We also noticed that the ratio between two isomers remains relatively unchanged for both GEC + 1 hydrolyzed linker-PL and SCDK + 1 hydrolyzed linker-PL across different time points.

The multiple peaks in Figure 3B,C can be explained by the constitutional isomers from thio-succinimide hydrolysis. The chirality of the α-carbon that connects the thiol and succinimide groups could also contribute to the observation of multiple peaks. More detailed structural assignments to each specific peak in Figure 3B,C are difficult due to the complexity of different potential isomers presenting on the THT peptide and would require additional experimentation.

### Identification of Linker-Payload Deconjugation and the Resulting Disulfide Bond Formation between Antibody and Endogenous Thiol-Bearing Molecules

The thio-succinimide hydrolysis product is not susceptible to linker-PL deconjugation, and our bottom-up data demonstrated that linker-PL ring-opening hydrolysis is the dominant reaction, consistent with our previous observations using LBA-LC-HRMS explaining AZD8205 stability.^35^ However, a very minor pool of AZD8205 undergoes deconjugation of the linker-PL. This biotransformation reaction then leads to cysteine and GSH adducts by disulfide bond formation with liberated thiol. Using a LBA-LC-MRM^HR^ approach, we confirmed the presence of Cys and GSH adducts on the Hc of AZD8205.

**Figure 4:**
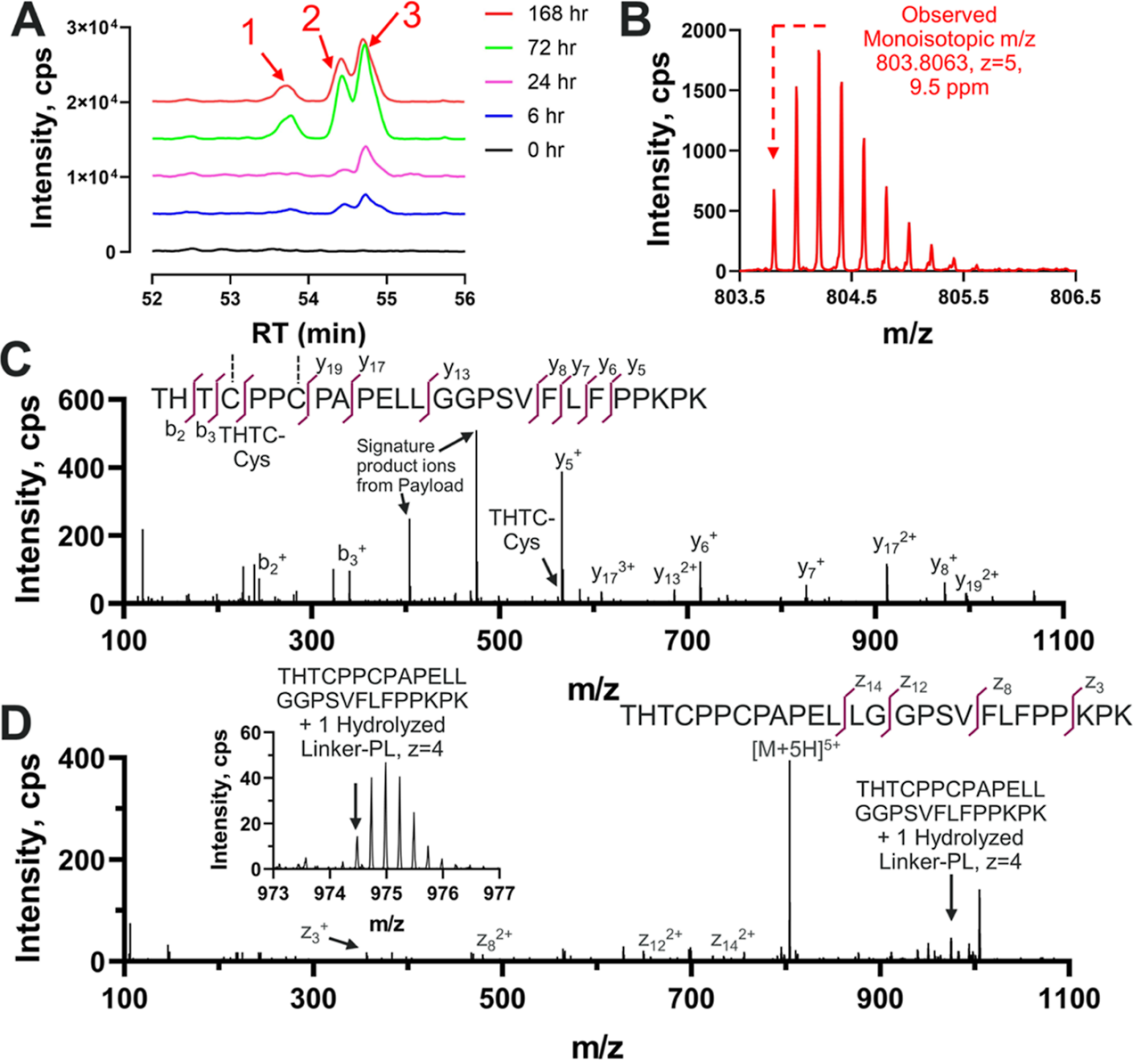
Formation of free cysteine adduct after linker-PL deconjugation at the Hc hinge region. This biotransformation species is noted as THT Cys adduct. (A) XICs of THT Cys adduct at different time points. The XICs from MS^1^ were extracted using its theoretical monoisotopic *m*/*z* of 803.7987 and a 10 ppm window. Arrows indicating peaks (1, 2, and 3) with different RT are isomers of the same biotransformation product, (B) MS^1^ spectrum of THT Cys adduct after incubation for 168 h. Dashed arrow points to the monoisotopic *m*/*z* of this biotransformed species. Structural elucidation of the THT Cys adduct product in MS/MS spectrum using (C) CID or (D) EAD. Embedded subfigure in (D) is a zoomed-in MS/MS spectrum of THT + 1 hydrolyzed linker-PL product ion. The *b*/*y* and *z* ions are labeled on THT peptide. Signature product ions from the PL have theoretical monoisotopic *m*/*z* values of 404.1610 and 475.1981, both with a charge state of 1.

Figure 4A,B show the XICs and mass spectrum of THT peptide Cys adduct. This biotransformed species originates from one linker-PL deconjugation followed by Cys adduct formation, whereas the other conjugated linker-PL hydrolyzes. THT Cys adduct was not detected at 0 h, and its concentration increased with longer incubation time, as shown in Figure 4A. The mass spectrum in Figure 4B confirms the identity of THT Cys adduct at the MS^1^ level. At 168 h, there are mainly three peaks (labeled as 1, 2, and 3) observed in the XIC of THT Cys adduct biotransformation species. We hypothesize that they might originate from the deconjugation of both linker-PLs in the THT peptide followed by the formation of the Cys adduct, while the other linker-PL hydrolysis generates two constitutional isomers discussed previously. Figures 4C,D show CID and EAD MS/MS spectra of THT Cys adduct product (RT: 53.4–55.2 min). CID MS/MS spectrum shows multiple *b* and *y* ions matching THT peptide and linker-PL fragments. In particular, a THTC-Cys product ion is observed in Figure 4C, validating our hypothesis of the biotransformation of linker-PL deconjugation followed by cysteinylation. Furthermore, the EAD MS/MS spectrum provides additional evidence on THT Cys adduct formation with a unique peak representing THT + 1 hydrolyzed linker-PL being displayed in Figure 4D. By comparing the CID spectra (Figure S2A–C) of each individual peak in XICs (peaks 1, 2, and 3), we noticed the heterogeneity of cysteinylation on the THT peptide. However, the EAD spectra from three peaks did not indicate clear differences (Figure S2D–F).

Similarly, we also observed the formation of the THT GSH adduct. XICs across different time points and mass spectrum at 168 h are shown in Figure S3A,B, respectively. Interestingly, we observed four peaks with different RT for the THT GSH adduct in XIC. The CID MS/MS spectrum in Figure S3C confirms the glutathionylation of this biotransformed product, where a peak representing THTC-GSH was found in the MS/MS spectrum using CID. Similarly, we discovered a unique peak representing THT + 1 hydrolyzed linker-PL in the EAD MS/MS spectrum, as shown in Figure S3D. Within the family of reactive circulating thiol-containing molecules, we also observed homocysteine and Cys–Gly adduct formation on THT peptide backbone from Hc at a very low concentration (not shown). Hence, it is possible that other than confirmed adduct species (Cys, GSH, homoCys, and Cys–Gly), additional molecules bearing free thiol groups may react with liberated cysteines from linker-PL deconjugation. To our surprise, we did not observe Cys or GSH adduct formation on GEC and SCDK peptides, possibly due to low abundance, small size and/or hydrophilic nature of these adducts, preventing their effective detection using the current approach.

### LC-MRM^HR^ Characterizes Cysteine and GSH Adduct Sites

Average MS^2^ spectra in Figure 4C,D show *b*/*y*, *z* product ions and revealed the identity of thiol adduct groups, establishing the structural information on THT Cys/GSH adduct biotransformation products first. Then, intrigued by the observation of multiple peaks in XICs of THT Cys and GSH adducts, we further exploited the MS^2^ spectra from each XIC peak and investigated the possible positional isomerism of these products. It is worth noting that the THT peptide from the Hc hinge region has two linker-PL conjugation sites, C_4_ and C_7_, as shown in Figure 2A.

Figure S4A–C show the zoomed-in CID spectra at *m*/*z* 560–566 range for peaks 1, 2, and 3 (RT: 53.4–54.0, 54.2–54.5, and 54.5–55.2, respectively), as labeled in Figure 4A. By comparing CID spectra of THT Cys adduct from peaks 1–3, we were only able to find THTC-Cys product ion in peaks 2 and 3. Thus, peaks 2 and 3 correspond to the Cys adducts at C_4_ on the THT peptide, whereas peak 1 is the cysteinylation at C_7_ on the THT peptide. We hypothesize that the separation of peaks 2 and 3 likely originates from the thio-succinimide hydrolysis at the C_7_ position on the THT peptide.

**Figure 5:**
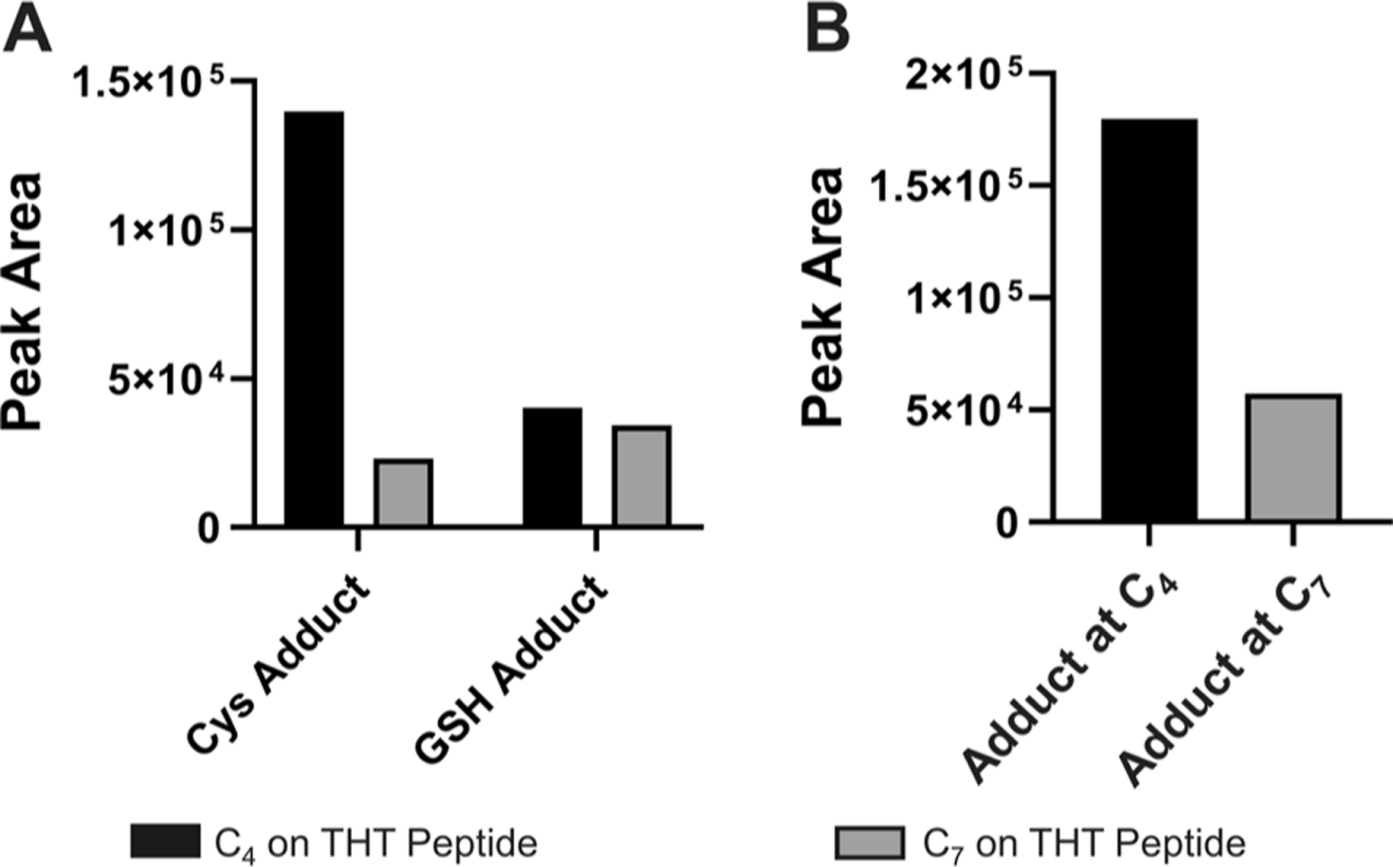
Relative peak areas of the formation of site-specific Cys or GSH adduct on either of the linker-PL conjugation sites on the Hc hinge region. (A) Distribution of Cys or GSH adduct at C_4_ or C_7_ on THT peptide (B) total peak area of thiol-adduct biotransformation species on the Hc hinge region. We only consider Cys and GSH in this case as the other thiol adducts (HomoCys, Cys–Gly etc.) have minimal peak area. The list of masses used for XIC is given in Table S2. The peak areas of XICs of MS^1^ were obtained by integrating AUC through MQ4 function in SCIEX MultiQuant.

We observed similar findings for the THT GSH adduct. We discovered the THTC-GSH product ion in the CID spectra of THT GSH adduct from peak 4, as shown in Figure S4G. Thus, peak 4 corresponds to the GSH adduct at C_4_ on THT peptide, whereas peaks 1, 2, and 3 correspond to the GSH adduct at C_7_ on THT peptide. By comparing the AUC of each peak in the XICs for both THT Cys and GSH adduct, we found that endogenous Cys shows strong preference for forming adduct at the C_4_ site on THT peptide of Hc, as indicated in Figure 5A. However, GSH does not appear to show a clear site preference for the adduct formation. Summing the AUCs of all THT Cys and GSH adducts together, we discovered that the C_4_ site shows a higher possibility of forming a thiol group adduct (Figure 5B).

### Characterization of the Antibody Interchain Disulfide Bond Formation after Linker-Payload Deconjugation

The exposed free thiol group generated from linker-PL deconjugation can undergo several biotransformation reactions.^12^ Other than forming disulfide bond with endogenous thiol-bearing molecules (e.g., cysteine, GSH, homocysteine, and Cys–Gly, etc.) from plasma, we also observed the disulfide bond reformation between Lc and Hc through intact mass LC-HRMS.^35^ Due to the conjugation strategy, there should be no interchain disulfide bonds present in AZD8205. The observation of the disulfide linked Lc + Hc product suggests that upon deconjugation, liberated cysteine can form an interchain disulfide bond. Here, we aim to confirm the presence of the biotransformation product that contains disulfide bond between Lc and Hc by using a bottom-up LC-MRM^HR^ approach. We also explored the possibility of disulfide linking between other antibody subunits.

**Figure 6:**
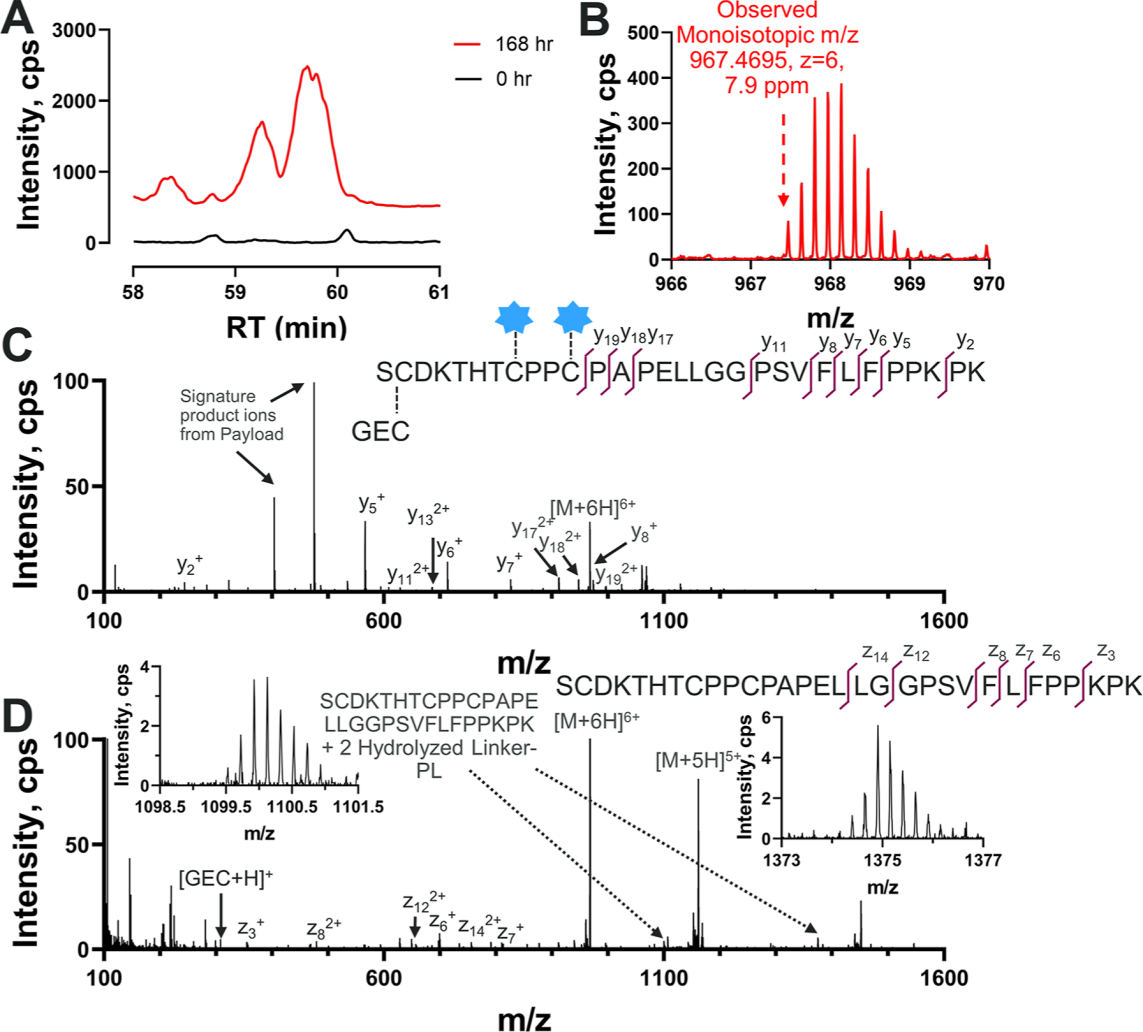
Disulfide bond reformation between Lc and Hc. This biotransformation species is noted as Lc + Hc. (A) XIC of MS1 Lc + Hc disulfide bond reformed biotransformation product. The XIC was extracted using monoisotopic *m*/*z* of 967.4619, and a 10 ppm window. (B) MS1 spectrum of Lc + Hc at 168 h. Dashed arrow points to the monoisotopic *m*/*z* of this biotransformation species. Structural elucidation of the Lc + Hc product in MS/MS spectra using (C) CID or (D) EAD. The blue stars are hydrolyzed linker-PLs. Embedded subfigures in (D) are zoomed-in MS/MS spectra of SCDKTHT + 2 hydrolyzed linker-PLs product ions at different charge states. The *y* and *z* ions are labeled as in SCDKTHT peptide.

Figure 6A shows the XICs of the Lc + Hc disulfide-linked biotransformation product after tryptic digestion. The sequence of this product is GE***C***-S***C***DKTHTCPPCPAPELLGGPSVFLFPPKPK (the hydrolyzed linker-PLs were conjugated on CPPC with the first two cysteines linked via a disulfide bond). We hypothesize that the reason for the missed cleavage was because trypsin was less accessible to the antibody backbone in close proximity to the linker-PL conjugation.^51^ While the product with no missed cleavage theoretically should be present as well, it cannot be identified through our current method, possibly due to its low-molecular weight and strong hydrophilicity. Alternatively, the missed cleavage could be the dominant species. By comparison of the XICs at different incubation times, the Lc + Hc structure was not present in samples without incubation, and its concentration increased with incubation time. Thus, Lc + Hc was confirmed to be a biotransformation product. Figure 6C shows the CID MS/MS spectrum of this product. The labeled *b* and *y* ions were from the SCDKTHTCPPCPAPELLGGPSVFLFPPKPK peptide (SCDKTHT peptide), suggesting high sequence coverage. The EAD MS/MS spectrum also provided additional evidence including individual GEC and SCDKTHT + 2 hydrolyzed linker-PL product ions (in Figure 6D). The LC-MRM^HR^ approach enabled both the confirmation of the disulfide bond reformation between Lc and Hc and the elucidation of the sites involved using CID and EAD.

Similarly, we observed the disulfide bond reformation between two Hc. This reformed disulfide bond is located between the opposing THT sites from each Hc, with one linker-PL on each Hc attached via a hydrolyzed thio-succinimide linker still remaining. Due to the low concentration of this biotransformed species, the XICs over different incubation times are not shown here. Figure S5A shows the mass spectrum of the disulfide bond linking two Hc species at 0 and 168 h, suggesting that this species is not endogenous. The CID MS/MS spectrum confirms the sequence coverage of the THT peptide backbone, as shown in Figure S5B. However, we were not able to assign the disulfide locations to specific cysteines from THT peptide due to extremely low abundance of this species. EAD MS/MS spectrum in Figure S5C shows a distinct product ion, representing THT + 1 hydrolyzed linker-PL. This information reveals further details of the structure of the Hc + Hc biotransformation species. Previous biotransformation research on AZD8205 using intact mass LC-HRMS revealed that over 90% of AZD8205 went through linker hydrolysis, stabilizing the PL conjugation, with the rest portion of ADC experiencing various biotransformations.^35^ For instance, the species with Lc + Hc disulfide reformed only accounts for approximately 2.5% of the whole pool of ADC. However, the Hc + Hc disulfide reformed species has even lower concentration and was not distinguishable using the intact mass method, further highlighting the high sensitivity of the LBA-LC-MRM^HR^ approach employed.

We also observed an intrachain disulfide bond formation between two adjacent cysteines within THT peptide (Figure S6). The XICs from 0 and 168 h incubation time confirm that this product was formed during the incubation, as shown in Figure S6A. Figure S6B confirms the identity of this biotransformation product at the MS level. The CID MS/MS spectrum shows high sequence coverage of this product, as shown in Figure S6C. *c* and *z* ions were labeled in the EAD MS/MS spectrum in Figure S6D. We noticed a couple of product ions (red color *b* and *c* ions in Figure S5C,D) that have observed masses that are ∼2 Da less than their theoretical values. These ions further confirm the formation of intrachain disulfide bond. Interestingly, in Figure S5D, the ion with disulfide bond dissociated overlapped with the nondissociated precursor ion, possibly because there was no mass change for this structure upon applying EAD. We hypothesize that the formation of the intrachain disulfide bond stems from the fact that two cysteines are spatially close to each other.

## Conclusions

In conclusion, we employed tryptic digestion and LBA-LC-MRM^HR^ approaches with two orthogonal dissociation methods: CID and EAD to confirm and further characterize our previous findings on AZD8205 biotransformation. Overall, bottom-up LC-MS/MS not only enabled the identification of low concentration biotransformation products but also provided further structural details. By applying CID, we characterized the progression of thio-succinimide hydrolysis over time. The obtained results align with the analyses performed using a previously reported intact protein LBA-LC-HRMS method. This study once again confirmed that the structural stability of AZD8205 originated from the high degree of thio-succinimide linker hydrolysis. The formation of the thiol group adduct on the antibody could be possibly explained by the Retro-Michael reaction liberating thiol groups, then leading to disulfide linkage with endogenous or neighboring thiols. Besides, CID aided in the investigation of the site preference of Cys or the GSH adduct on Hc. We noticed that the C_4_ on THT peptide at the Hc hinge region showed a slightly higher possibility of linker-PL deconjugation followed by forming thiol adduct. Additionally, we found disulfide reformation between antibody subunits and the formation of an intrachain disulfide bond on Hc. We also highlight the application of EAD for providing additional details in the characterization of biotransformation species. Unique diagnostic ions generated from EAD supported the differentiation of constitutional isomers from thio-succinimide hydrolysis. EAD also provided additional evidence on structural elucidation of disulfide linked products by preferentially cleaving disulfide bonds while leaving the peptide backbone intact. EAD provided sufficient information for the structural elucidation of these biotransformed species under current conditions. However, the parameters could be optimized to offer better dissociation efficiency, potentially enabling the assignment of thio-Asp and thio-isoAsp isomers on the THT peptide. The LC-MRM^HR^ approach with orthogonal dissociation methods offered complementary information for characterizing AZD8205 biotransformation products in detail. This work, in concert with other studies of ADC biotransformation, creates the necessary scholarship for future implementation of knowledge-based biotransformation pathways predicted by software coupled with LC-MRM^HR^ analyses to examine a much wider range of potential biotransformation species.^52^ These analyses can uncover further structural changes for ADCs *in vivo*, helping scientists better understand their stability determinants and PK profiles.

## Supporting Information Available

Structures, monoisotopic *m*/*z* values of biotransformation products, relative quantification of constitutional and positional isomers, and XIC, MS, and MS^2^ spectra of biotransformation species (PDF)

## References

[ref1] Dumontet C.; Reichert J. M.; Senter P. D.; Lambert J. M.; Beck A. Antibody-Drug Conjugates Come of Age in Oncology. Nat. Rev. Drug Discovery 2023, 22 (8), 641–661. 10.1038/s41573-023-00709-2. 37308581

[ref2] Drago J. Z.; Modi S.; Chandarlapaty S. Unlocking the Potential of Antibody-Drug Conjugates for Cancer Therapy. Nat. Rev. Clin. Oncol. 2021, 18 (6), 327–344. 10.1038/s41571-021-00470-8. 33558752 PMC8287784

[ref3] do Pazo C.; Nawaz K.; Webster R. M. The Oncology Market for Antibody-Drug Conjugates. Nat. Rev. Drug Discovery 2021, 20 (8), 583–584. 10.1038/d41573-021-00054-2. 33762691

[ref4] Jabbour E.; Paul S.; Kantarjian H. The Clinical Development of Antibody-Drug Conjugates - Lessons from Leukaemia. Nat. Rev. Clin. Oncol. 2021, 18 (7), 418–433. 10.1038/s41571-021-00484-2. 33758376

[ref5] Jin Y.; Schladetsch M. A.; Huang X.; Balunas M. J.; Wiemer A. J. Stepping forward in antibody-drug conjugate development. Pharmacol. Ther. 2022, 229, 107917 10.1016/j.pharmthera.2021.107917. 34171334 PMC8702582

[ref6] Garcia-Alonso S.; Ocana A.; Pandiella A. Trastuzumab Emtansine: Mechanisms of Action and Resistance, Clinical Progress, and Beyond. Trends Cancer 2020, 6 (2), 130–146. 10.1016/j.trecan.2019.12.010. 32061303

[ref7] Yaghoubi S.; Karimi M. H.; Lotfinia M.; Gharibi T.; Mahi-Birjand M.; Kavi E.; Hosseini F.; Sineh Sepehr K.; Khatami M.; Bagheri N.; Abdollahpour-Alitappeh M. Potential drugs used in the antibody-drug conjugate (ADC) architecture for cancer therapy. J. Cell. Physiol. 2020, 235 (1), 31–64. 10.1002/jcp.28967. 31215038

[ref8] Matsuda Y.; Seki T.; Yamada K.; Ooba Y.; Takahashi K.; Fujii T.; Kawaguchi S.; Narita T.; Nakayama A.; Kitahara Y.; Mendelsohn B. A.; et al. Chemical Site-Specific Conjugation Platform to Improve the Pharmacokinetics and Therapeutic Index of Antibody-Drug Conjugates. Mol. Pharmaceutics 2021, 18 (11), 4058–4066. 10.1021/acs.molpharmaceut.1c00473. 34579528

[ref9] Kramlinger V. M.; Dalvie D.; Heck C. J. S.; Kalgutkar A. S.; O’Neill J.; Su D.; Teitelbaum A. M.; Totah R. A. Future of Biotransformation Science in the Pharmaceutical Industry. Drug Metab. Dispos. 2022, 50 (3), 258–267. 10.1124/dmd.121.000658. 34921097

[ref10] Jian W.; Wei C.; Gan a. J. Bioanalytical Aspects in Biological Therapeutics. In Bioanalytical Aspects in Biological Therapeutics; Wiley, 2022; pp 277–307.

[ref11] Walles M.; Berna M.; Jian W.; Hauri S.; Hengel S.; King L.; Tran J. C.; Wei C.; Xu K.; Zhu X. A Cross Company Perspective on the Assessment of Therapeutic Protein Biotransformation. Drug Metab. Dispos. 2022, 50 (6), 846–857. 10.1124/dmd.121.000462. 35306476

[ref12] Su D.; Kozak K. R.; Sadowsky J.; Yu S. F.; Fourie-O’Donohue A.; Nelson C.; Vandlen R.; Ohri R.; Liu L.; Ng C.; He J.; Davis H.; Lau J.; Del Rosario G.; Cosino E.; Cruz-Chuh J. d.; Ma Y.; Zhang D.; Darwish M.; Cai W.; Chen C.; Zhou H.; Lu J.; Liu Y.; Kaur S.; Xu K.; Pillow T. H. Modulating Antibody-Drug Conjugate Payload Metabolism by Conjugation Site and Linker Modification. Bioconjugate Chem. 2018, 29 (4), 1155–1167. 10.1021/acs.bioconjchem.7b00785. 29481745

[ref13] Su D.; Ng C.; Khosraviani M.; Yu S. F.; Cosino E.; Kaur S.; Xu K. Custom-Designed Affinity Capture LC-MS F(ab’)2 Assay for Biotransformation Assessment of Site-Specific Antibody Drug Conjugates. Anal. Chem. 2016, 88 (23), 11340–11346. 10.1021/acs.analchem.6b03410. 27779866

[ref14] Huang Y.; Mou S.; Wang Y.; Mu R.; Liang M.; Rosenbaum A. I. Characterization of Antibody-Drug Conjugate Pharmacokinetics and in Vivo Biotransformation Using Quantitative Intact LC-HRMS and Surrogate Analyte LC-MRM. Anal. Chem. 2021, 93 (15), 6135–6144. 10.1021/acs.analchem.0c05376. 33835773

[ref15] Shen B. Q.; Xu K.; Liu L.; Raab H.; Bhakta S.; Kenrick M.; Parsons-Reponte K. L.; Tien J.; Yu S. F.; Mai E.; Li D.; Tibbitts J.; Baudys J.; Saad O. M.; Scales S. J.; McDonald P. J.; Hass P. E.; Eigenbrot C.; Nguyen T.; Solis W. A.; Fuji R. N.; Flagella K. M.; Patel D.; Spencer S. D.; Khawli L. A.; Ebens A.; Wong W. L.; Vandlen R.; Kaur S.; Sliwkowski M. X.; Scheller R. H.; Polakis P.; Junutula J. R. Conjugation site modulates the in vivo stability and therapeutic activity of antibody-drug conjugates. Nat. Biotechnol. 2012, 30 (2), 184–189. 10.1038/nbt.2108. 22267010

[ref16] Su D.; Zhang D. Linker Design Impacts Antibody-Drug Conjugate Pharmacokinetics and Efficacy via Modulating the Stability and Payload Release Efficiency. Front. Pharmacol 2021, 12, 687926 10.3389/fphar.2021.687926. 34248637 PMC8262647

[ref17] He J.; Su D.; Ng C.; Liu L.; Yu S. F.; Pillow T. H.; Del Rosario G.; Darwish M.; Lee B. C.; Ohri R.; Zhou H.; Wang X.; Lu J.; Kaur S.; Xu K. High-Resolution Accurate-Mass Mass Spectrometry Enabling In-Depth Characterization of in Vivo Biotransformations for Intact Antibody-Drug Conjugates. Anal. Chem. 2017, 89 (10), 5476–5483. 10.1021/acs.analchem.7b00408. 28429938

[ref18] Mu R.; Yuan J.; Huang Y.; Meissen J. K.; Mou S.; Liang M.; Rosenbaum A. I. Bioanalytical Methods and Strategic Perspectives Addressing the Rising Complexity of Novel Bioconjugates and Delivery Routes for Biotherapeutics. BioDrugs 2022, 36 (2), 181–196. 10.1007/s40259-022-00518-w. 35362869 PMC8972746

[ref19] Tumey L. N.; Charati M.; He T.; Sousa E.; Ma D.; Han X.; Clark T.; Casavant J.; Loganzo F.; Barletta F.; Lucas J.; Graziani E. I. Mild Method for Succinimide Hydrolysis on ADCs: Impact on ADC Potency, Stability, Exposure, and Efficacy. Bioconjugate Chem. 2014, 25 (10), 1871–1880. 10.1021/bc500357n. 25216346

[ref20] Shi C.; Goldberg S.; Lin T.; Dudkin V.; Widdison W.; Harris L.; Wilhelm S.; Jmeian Y.; Davis D.; O’Neil K.; Weng N.; Jian W. LC/MS/MS Bioanalysis of Protein-Drug Conjugates-The Importance of Incorporating Succinimide Hydrolysis Products. Anal. Chem. 2018, 90 (8), 5314–5321. 10.1021/acs.analchem.8b00411. 29589741

[ref21] Kallsten M.; Hartmann R.; Artemenko K.; Lind S. B.; Lehmann F.; Bergquist J. Qualitative Analysis of Antibody-Drug Conjugates (ADCs): an Experimental Comparison of Analytical Techniques of Cysteine-Linked ADCs. Analyst 2018, 143 (22), 5487–5496. 10.1039/C8AN01178H. 30289422

[ref22] Holte D.; Lyssikatos J. P.; Valdiosera A. M.; Swinney Z.; Sisodiya V.; Sandoval J.; Lee C.; Aujay M. A.; Tchelepi R. B.; Hamdy O. M.; Gu C.; Lin B.; Sarvaiya H.; Pysz M. A.; Laysang A.; Williams S.; Lee D. J.; Holda M. K.; Purcell J. W.; Gavrilyuk J. Evaluation of PNU-159682 Antibody Drug Conjugates (ADCs). Bioorg. Med. Chem. Lett. 2020, 30 (24), 127640 10.1016/j.bmcl.2020.127640. 33127540

[ref23] Huang Y.; Yuan J.; Mu R.; Kubiak R. J.; Ball K.; Cao M.; Hussmann G. P.; de Mel N.; Liu D.; Roskos L. K.; Liang M.; Rosenbaum A. I. Multiplex Bioanalytical Methods for Comprehensive Characterization and Quantification of the Unique Complementarity-Determining-Region Deamidation of MEDI7247, an Anti-ASCT2 Pyrrolobenzodiazepine Antibody-Drug Conjugate. Antibodies 2023, 12 (4), 66 10.3390/antib12040066. 37873863 PMC10594446

[ref24] Cao M.; Hussmann G. P.; Tao Y.; O’Connor E.; Parthemore C.; Zhang-Hulsey D.; Liu D.; Jiao Y.; Mel N.; Prophet M.; Korman S.; Sonawane J.; Grigoriadou C.; Huang Y.; Umlauf S.; Chen X. Atypical Asparagine Deamidation of NW Motif Significantly Attenuates the Biological Activities of an Antibody Drug Conjugate. Antibodies 2023, 12 (4), 68 10.3390/antib12040068. 37987246 PMC10660493

[ref25] Nguyen T. D.; Bordeau B. M.; Balthasar J. P. Mechanisms of ADC Toxicity and Strategies to Increase ADC Tolerability. Cancers 2023, 15 (3), 713 10.3390/cancers15030713. 36765668 PMC9913659

[ref26] Chang H.-P.; Shah D. K. Determination of ADC Concentration by Ligand-Binding Assays. In Methods in Molecular Biology; Springer, 2019; Vol. 2078; pp 361–369, 10.1007/978-1-4939-9929-3_26. 31643071

[ref27] Wang J.; Gu H.; Liu A.; Kozhich A.; Rangan V.; Myler H.; Luo L.; Wong R.; Sun H.; Wang B.; Vezina H. E.; Deshpande S.; Zhang Y.; Yang Z.; Olah T. V.; Aubry A.-F.; Arnold M. E.; Pillutla R.; DeSilva B. Antibody-Drug Conjugate Bioanalysis Using LB-LC-MS/MS Hybrid Assays: Strategies, Methodology and Correlation to Ligand-Binding Assays. Bioanalysis 2016, 8 (13), 1383–1401. 10.4155/bio-2016-0017. 27277879

[ref28] Schadt S.; Hauri S.; Lopes F.; Edelmann M. R.; Staack R. F.; Villasenor R.; Kettenberger H.; Roth A. B.; Schuler F.; Richter W. F.; Funk C. Are Biotransformation Studies of Therapeutic Proteins Needed? Scientific Considerations and Technical Challenges. Drug Metab. Dispos. 2019, 47 (12), 1443–1456. 10.1124/dmd.119.088997. 31748266

[ref29] Mou S.; Huang Y.; Rosenbaum A. I. ADME Considerations and Bioanalytical Strategies for Pharmacokinetic Assessments of Antibody-Drug Conjugates. Antibodies 2018, 7 (4), 41 10.3390/antib7040041. 31544891 PMC6698957

[ref30] Mu R.; Huang Y.; Bouquet J.; Yuan J.; Kubiak R. J.; Ma E.; Naser S.; Mylott W. R.; Ismaiel O. A.; Wheeler A. M.; Burkart R.; Cortes D. F.; Bruton J.; Arends R. H.; Liang M.; Rosenbaum A. I. Multiplex Hybrid Antigen-Capture LC-MRM Quantification in Sera and Nasal Lining Fluid of AZD7442, a SARS-CoV-2-Targeting Antibody Combination. Anal. Chem. 2022, 94 (43), 14835–14845. 10.1021/acs.analchem.2c01320. 36269894 PMC9631352

[ref31] Huang Y.; Del Nagro C. J.; Balic K.; Mylott W. R.; Ismaiel O. A.; Ma E.; Faria M.; Wheeler A. M.; Yuan M.; Waldron M. P.; Peay M. G.; Cortes D. F.; Roskos L.; Liang M.; Rosenbaum A. I. Multifaceted Bioanalytical Methods for the Comprehensive Pharmacokinetic and Catabolic Assessment of MEDI3726, an Anti-Prostate-Specific Membrane Antigen Pyrrolobenzodiazepine Antibody-Drug Conjugate. Anal. Chem. 2020, 92 (16), 11135–11144. 10.1021/acs.analchem.0c01187. 32459957

[ref32] Pandey R.; Gruslova A.; Chiou J.; Brenner A. J.; Tiziani S. Stable Isotope Dilution LC-HRMS Assay To Determine Free SN-38, Total SN-38, and SN-38G in a Tumor Xenograft Model after Intravenous Administration of Antibody-Drug Conjugate (Sacituzumab Govitecan). Anal. Chem. 2020, 92 (1), 1260–1267. 10.1021/acs.analchem.9b04419. 31765123

[ref33] Jashnani A.; Kotapati S.; Deshpande M.; Yamazoe S.; Strop P.; Rajpal A.; Dollinger G. Automated and Faster Affinity Capture Method for Biotransformation Assessment of Site-Specific Antibody Drug Conjugates. Anal. Chem. 2021, 93 (13), 5371–5376. 10.1021/acs.analchem.0c04685. 33750099

[ref34] Kotapati S.; Passmore D.; Yamazoe S.; Sanku R. K. K.; Cong Q.; Poudel Y. B.; Chowdari N. S.; Gangwar S.; Rao C.; Rangan V. S.; Cardarelli P. M.; Deshpande S.; Strop P.; Dollinger G.; Rajpal A. Universal Affinity Capture Liquid Chromatography-Mass Spectrometry Assay for Evaluation of Biotransformation of Site-Specific Antibody Drug Conjugates in Preclinical Studies. Anal. Chem. 2020, 92 (2), 2065–2073. 10.1021/acs.analchem.9b04572. 31860282

[ref35] Huang Y.; Tan H. Y.; Yuan J.; Mu R.; Yang J.; Ball K.; Vijayakrishnan B.; Masterson L.; Kinneer K.; Luheshi N.; Liang M.; Rosenbaum A. I. Extensive Biotransformation Profiling of AZD8205, an Anti-B7-H4 Antibody-Drug Conjugate, Elucidates Pathways Underlying its Stability In Vivo. Anal. Chem. 2024, 96, 16525–16533. 10.1021/acs.analchem.4c02309. 39392424 PMC11503519

[ref36] Bults P.; Spanov B.; Olaleye O.; van de Merbel N. C.; Bischoff R. Intact protein bioanalysis by liquid chromatography - High-resolution mass spectrometry. J. Chromatogr. B Analyt. Technol. Biomed. Life Sci. 2019, 1110–1111, 155–167. 10.1016/j.jchromb.2019.01.032. 30849729

[ref37] Kellie J. F.; Pannullo K. E.; Li Y.; Fraley K.; Mayer A.; Sychterz C. J.; Szapacs M. E.; Karlinsey M. Z. Antibody Subunit LC-MS Analysis for Pharmacokinetic and Biotransformation Determination from In-Life Studies for Complex Biotherapeutics. Anal. Chem. 2020, 92 (12), 8268–8277. 10.1021/acs.analchem.0c00520. 32392410

[ref38] Kinneer K.; Wortmann P.; Cooper Z. A.; Dickinson N. J.; Masterson L.; Cailleau T.; Hutchinson I.; Vijayakrishnan B.; McFarlane M.; Ball K.; Davies M.; Lewis A.; Huang Y.; Rosenbaum A. I.; Yuan J.; Chesebrough J.; Anderton J.; Monks N.; Novick S.; Wang J.; Dimasi N.; Christie R. J.; Sabol D.; Tosto F. A.; Wallez Y.; Leo E.; Albertella M. R.; Staniszewska A. D.; Tice D. A.; Howard P. W.; Luheshi N.; Sapra P. Design and Preclinical Evaluation of a Novel B7-H4-Directed Antibody-Drug Conjugate, AZD8205, Alone and in Combination with the PARP1-Selective Inhibitor AZD5305. Clin. Cancer Res. 2023, 29 (6), 1086–1101. 10.1158/1078-0432.CCR-22-2630. 36355054

[ref39] Kellie J. F.; Thomson A. S.; Chen S.; Childs S. L.; Karlinsey M. Z.; Mai S. H.; White J. R.; Biddlecombe R. A. Biotherapeutic Antibody Subunit LC-MS and Peptide Mapping LC-MS Measurements to Study Possible Biotransformation and Critical Quality Attributes In Vivo. J. Pharm. Sci. 2019, 108 (4), 1415–1422. 10.1016/j.xphs.2018.11.019. 30465782

[ref40] Flasch M.; Koellensperger G.; Warth B. Comparing the Sensitivity of a Low- and a High-Resolution Mass Spectrometry Approach for Xenobiotic Trace Analysis: An Exposome-Type Case Study. Anal. Chim. Acta 2023, 1279, 341740 10.1016/j.aca.2023.341740. 37827628

[ref41] Wells J. M.; McLuckey S. A. Collision-Induced Dissociation (CID) of Peptides and Proteins. Methods Enzymol. 2005, 402, 148–185. 10.1016/S0076-6879(05)02005-7. 16401509

[ref42] Medzihradszky K. F.; Campbell J. M.; Baldwin M. A.; Falick A. M.; Juhasz P.; Vestal M. L.; Burlingame A. L. The Characteristics of Peptide Collision-Induced Dissociation using a High-Performance MALDI-TOF/TOF Tandem Mass Spectrometer. Anal. Chem. 2000, 72 (3), 552–558. 10.1021/ac990809y. 10695141

[ref43] Zhurov K. O.; Fornelli L.; Wodrich M. D.; Laskay U. A.; Tsybin Y. O. Principles of Electron Capture and Transfer Dissociation Mass Spectrometry Applied to Peptide and Protein Structure Analysis. Chem. Soc. Rev. 2013, 42 (12), 5014–5030. 10.1039/c3cs35477f. 23450212

[ref44] Beckman J. S.; Voinov V. G.; Hare M.; Sturgeon D.; Vasil’ev Y.; Oppenheimer D.; Shaw J. B.; Wu S.; Glaskin R.; Klein C.; Schwarzer C.; Stafford G. Improved Protein and PTM Characterization with a Practical Electron-Based Fragmentation on Q-TOF Instruments. J. Am. Soc. Mass Spectrom. 2021, 32 (8), 2081–2091. 10.1021/jasms.0c00482. 33914527 PMC8343505

[ref45] Liu F.; van Breukelen B.; Heck A. J. Facilitating Protein Disulfide Mapping by a Combination of Pepsin Digestion, Electron Transfer Higher Energy Dissociation (EThcD), and a Dedicated Search Algorithm SlinkS. Mol. Cell. Proteomics 2014, 13 (10), 2776–2786. 10.1074/mcp.O114.039057. 24980484 PMC4189002

[ref46] He Y.; Hou P.; Long Z.; Zheng Y.; Tang C.; Jones E.; Diao X.; Zhu M. Application of Electro-Activated Dissociation Fragmentation Technique to Identifying Glucuronidation and Oxidative Metabolism Sites of Vepdegestrant by Liquid Chromatography-High Resolution Mass Spectrometry. Drug Metab. Dispos. 2024, 52, 634–643. 10.1124/dmd.124.001661. 38830773

[ref47] Sun Z.; Huang M.; Sokolowska I.; Cao R.; Chang K.; Hu P.; Mo J. Impact of Trisulfide on the Structure and Function of Different Antibody Constructs. J. Pharm. Sci. 2023, 112 (10), 2637–2643. 10.1016/j.xphs.2023.08.010. 37595748

[ref48] Yang J.; Yuan J.; Huang Y.; Rosenbaum A. I. Reference-Free Thio-Succinimide Isomerization Characterization by Electron-Activated Dissociation. Rapid Commun. Mass Spectrom. 2024, 38, e9910 10.1002/rcm.9910. 39287024

[ref49] Geiger T.; Clarke S. Deamidation, Isomerization, and Racemization at Asparaginyl and Aspartyl Residues in Peptides. Succinimide-Linked Reactions that Contribute to Protein Degradation. J. Biol. Chem. 1987, 262 (2), 785–794. 10.1016/S0021-9258(19)75855-4. 3805008

[ref50] Yan Q.; Huang M.; Lewis M. J.; Hu P. Structure Based Prediction of Asparagine Deamidation Propensity in Monoclonal Antibodies. MAbs 2018, 10 (6), 901–912. 10.1080/19420862.2018.1478646. 29958069 PMC6152450

[ref51] Chen L.; Wang L.; Shion H.; Yu C.; Yu Y. Q.; Zhu L.; Li M.; Chen W.; Gao K. In-depth structural characterization of Kadcyla (ado-trastuzumab emtansine) and its biosimilar candidate. MAbs 2016, 8 (7), 1210–1223. 10.1080/19420862.2016.1204502. 27380163 PMC5058630

[ref52] Liu K.; Ai Y.; Tan H. Y.; Yuan J.; Meissen J. K.; Huang Y.; Rosenbaum A. I. High-Throughput Automated Data Analysis workflow for ADC Biotransformation Characterization. ChemRxiv 2024, 10.26434/chemrxiv-2024-lwpz2. PMC11948178 40066817

