# Supplementary material for: Detailed Structural Elucidation of Antibody-Drug Conjugate Biotransformation Species Using High Resolution Multiple Reaction Monitoring Mass Spectrometry with Orthogonal Dissociation Methods
Source: ACS Pharmacol Transl Sci. 2024 Dec 17;8(1):113–23. doi: 10.1021/acsptsci.4c00445 (PMC11729422; doi:10.1021/acsptsci.4c00445)
Supplement: Supplementary file 1 — pt4c00445_si_001.pdf [file pt4c00445_si_001.pdf]

Supporting Information

Detailed Structural Elucidation of Antibody-Drug Conjugate Biotransformation Species Using High Resolution Multiple Reaction Monitoring Mass Spectrometry with Orthogonal Dissociation Methods

Junyan Yang[1§], Hui Yin Tan[1], Jiaqi Yuan[1], Yue Huang[1††], and Anton I. Rosenbaum[1*††]

Affiliation:

[1]Integrated Bioanalysis, Clinical Pharmacology and Safety Sciences, R&D, AstraZeneca, South San Francisco, CA 94080, USA

*Corresponding Author:
Anton I. Rosenbaum - 121 Oyster Point Blvd, South San Francisco, CA 94080, USA; Tel: +1-650-379-3099; E-mail: anton.rosenbaum@astrazeneca.com, anton.rosenbaum.phd@gmail.com

Present Addresses:

[§]Junyan Yang - Eli Lilly and Company, 893 Delaware St, Indianapolis, IN 46225, USA. junyan.yang@lilly.com

[†]Yue Huang – Revolution Medicines, 700 Saginaw Drive, Redwood City, CA 94063, USA; Tel: +1-650-481-6801; E-mail: yhuang@revmed.com

[††]Anton Rosenbaum– Vera Therapeutics, 2000 Sierra Point Parkway, Brisbane, CA 94005, USA; anton.rosenbaum@veratx.com

Contents

S1. Summary of peptides containing non-hydrolyzed or hydrolyzed linker-payload …….……S-2

S2. Summary of identified biotransformation products and their composition through LC-MRM^{HR}………………………………………………………………………….....S-3

S3. Comparison of the ratio of thio-succinimide hydrolysis isomeric products from different linker-payload conjugation sites….……………………………………………………………S-5

S4. Full CID and EAD spectra of peak 1, 2 and 3 from THT Cys adduct………………………S-6

S5. Identification and structural elucidation of THT GSH adduct…………………………….....S-7

S6. Investigation of conjugation site-preference for forming disulfide bond with endogenous thiol-bearing molecules using CID fragmentation.……………..………………...………………..S-8

S7. Characterization of the disulfide bond reformation between two Hc using both CID and EAD fragmentation……………………………………………………………………………S-9

S8. Structural confirmation of an intra-chain disulfide-linked Hc biotransformation product...S-10

S1. Summary of peptides containing non-hydrolyzed or hydrolyzed linker-payload

Table S1. Composition of three different tryptic peptides containing non-hydrolyzed or hydrolyzed thio-succinimide linker-payload and their monoisotopic m/z. The yellow and blue stars are non-hydrolyzed and hydrolyzed Linker-PL, respectively. THT peptide has a full amino acid sequence of THTCPPCPAPELLGGPSVFLFPPKPK.

Species	Scheme	Structure	Charge State (z)	Theoretical Monoisotopic m/z
Lc + Linker-PL With or Without Hydrolysis	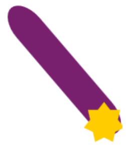	GEC + Linker-PL	3	485.8799
	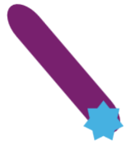	GEC + 1 Hydrolyzed Linker-PL	3	491.8834
Hc (above hinge) + Linker-PL With or Without Hydrolysis	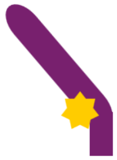	SCDK + Linker-PL	3	533.9099
	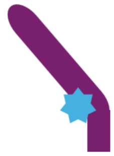	SCDK + 1 Hydrolyzed Linker-PL	3	539.9134
Hc (hinge region) + Linker-PL With Different Degree of Hydrolysis	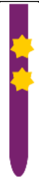	THT + 2 Linker-PLs	5	1005.9023
	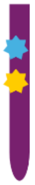 or 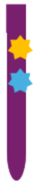	THT + 1 Linker-PL + 1 Hydrolyzed Linker-PL	5	1009.5044
	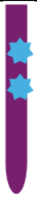	THT + 2 Hydrolyzed Linker-PLs	5	1013.1065

S2. Summary of identified biotransformation species and their composition through LC-MRM^{HR}

Table S2. Composition of three different tryptic peptides containing non-hydrolyzed or hydrolyzed thio-succinimide linker-payload and their monoisotopic m/z. The yellow and blue stars are non-hydrolyzed and hydrolyzed Linker-PL, respectively. Light and dark green circles are Cys and GSH, respectively. Orange and pink circles are HomoCys and Cys-Gly, respectively. SCDKTHT has a full amino acid sequence of SCDKTHTCPPCPAPELLGGPSVFLFPPKPK.

Species	Scheme	Structure	Charge State (z)	Theoretical Monoisotopic m/z
THT Cys Adduct on Hc hinge region	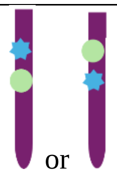	THT + 1 Hydrolyzed Linker-PL + 1 Cysteine	5	803.7987
THT GSH Adduct on Hc hinge region	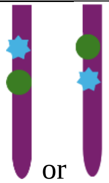	THT + 1 Hydrolyzed Linker-PL + 1 Glutathione	5	841.0115
THT HomoCys Adduct on Hc hinge region	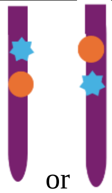	THT + 1 Hydrolyzed Linker-PL + 1 HomoCysteine	5	806.6019
THT Cys-Gly Adduct on Hc hinge region	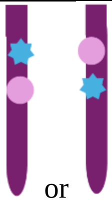	THT + 1 Hydrolyzed Linker-PL + 1 Cysteinylglycine	5	815.2030
Lc + Hc Inter-Chain Disulfide Bond Reformed	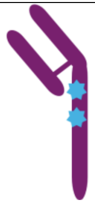	GEC + SCDKTHT + 2 Hydrolyzed Linker-PLs	6	967.4619

Hc + Hc Inter-Chain Disulfide Bond Reformed	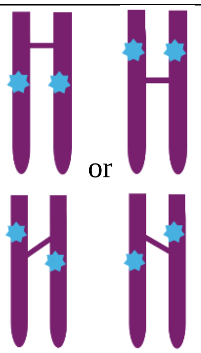	THT + 2 Hydrolyzed Linker-PLs	8	974.4935
Hc Intra-Chain Disulfide Bond Formed	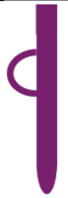	THT - 2 Hydrogen	4	682.8558

S3. Comparison of the ratios of thio-succinimide hydrolysis isomeric products from different linker-payload conjugation sites

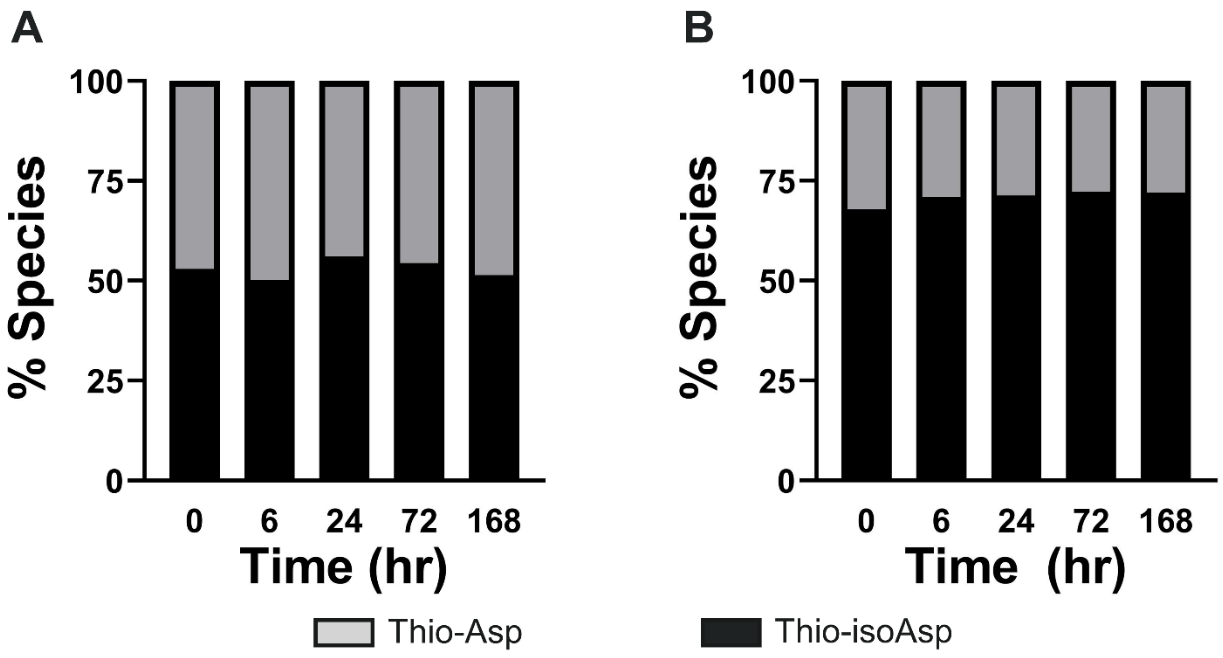

Figure S1. Comparison of the ratios of thio-succinimide hydrolysis isomeric products from different linker-payload conjugation sites. Relative abundances between thio-Asp and thio-isoAsp after thio-succinimide hydrolysis from (A) GEC peptide, (B) SCDK peptide.

S4. Full CID and EAD spectra of peak 1, 2 and 3 from THT Cys adduct

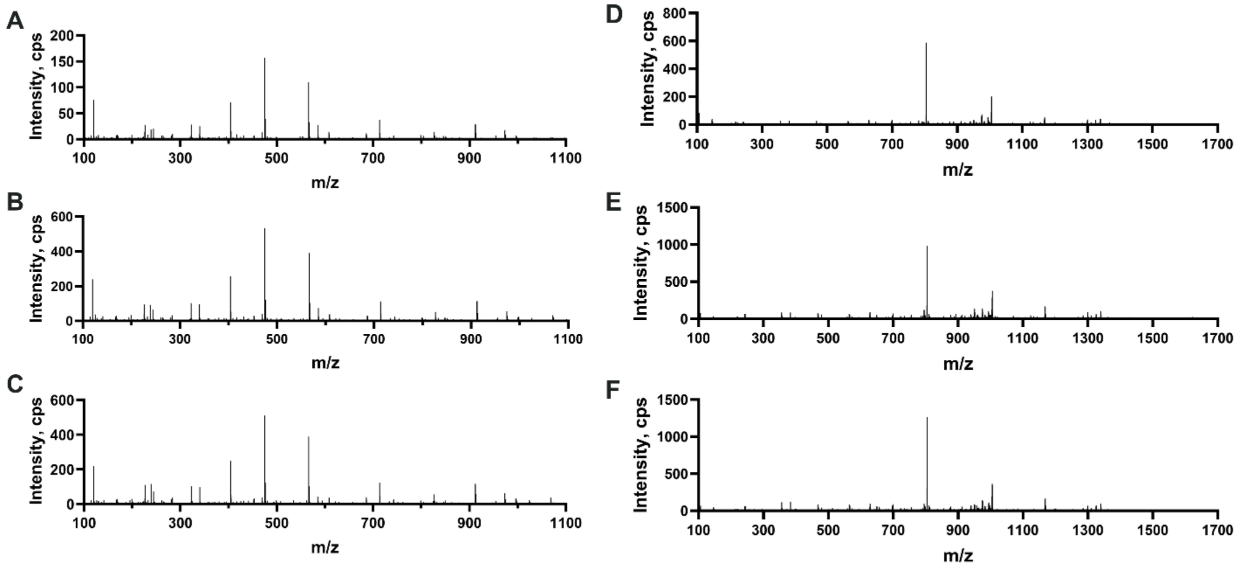

Figure S2. Full (A-C) CID and (D-F) EAD spectra of peak 1, 2, and 3 of THT Cys adduct. The chromatographic separation of three peaks in XIC indicates the heterogeneity of this biotransformation species. However, the full CID and EAD spectra from three peaks provided almost identical information. The differentiation of these three peaks requires careful investigation of zoomed-in spectra (see Figure S4).

S5. Identification and structural elucidation of THT GSH adduct

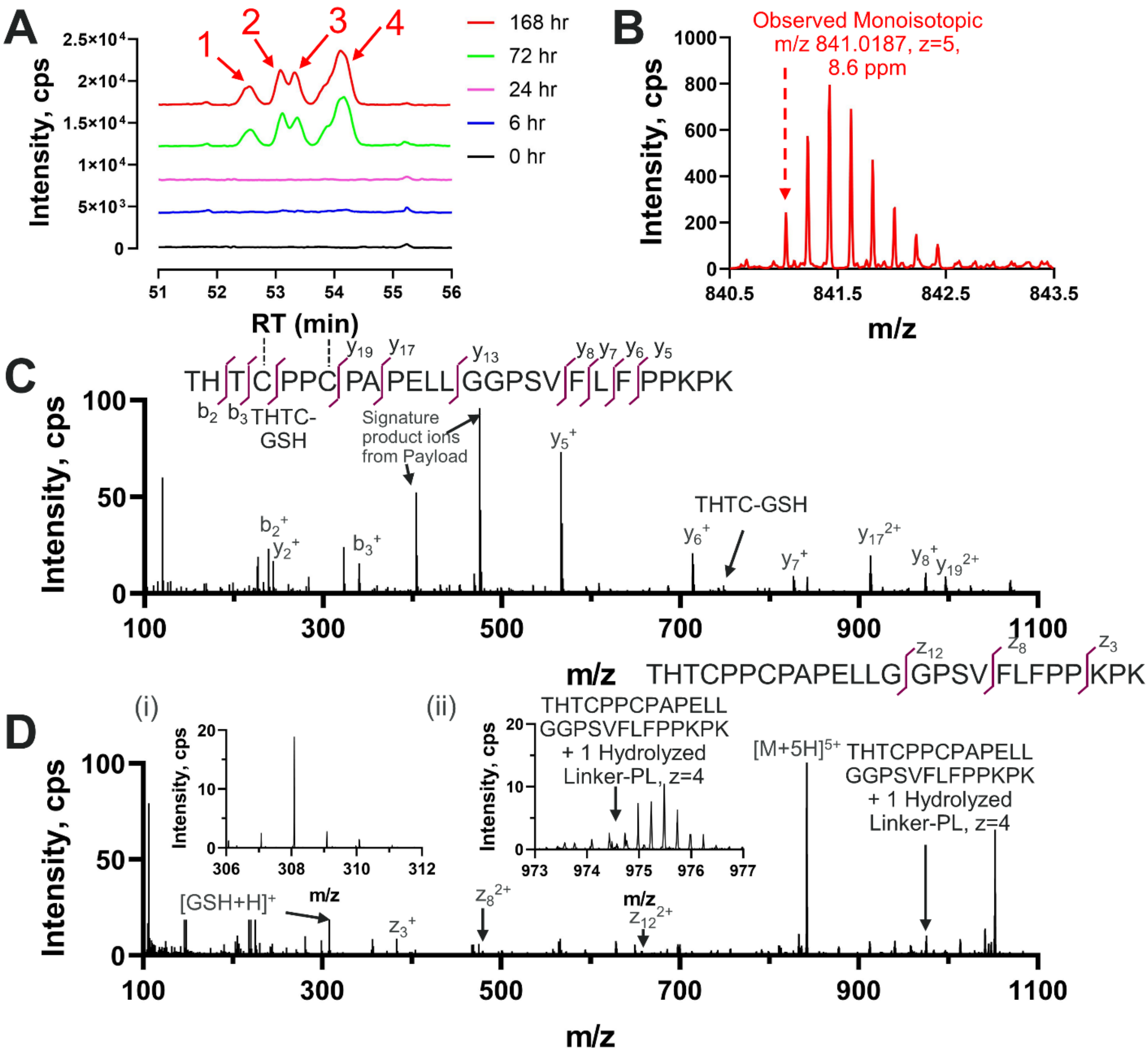

Figure S3. Formation of free GSH adduct after linker-PL deconjugation on Hc hinge region. This biotransformation species is noted as THT GSH adduct. (A) XIC of THT GSH adduct biotransformation product at different timepoints, with arrows denoting different isomer peaks (1, 2, 3 and 4) of the same biotransformation product (see section S6 for isomer differentiation results). XICs were extracted using theoretical monoisotopic m/z 841.0115, z=5 and a 10-ppm window. (B) MS spectrum of THT GSH adduct at 168 h. Dashed arrow points to its monoisotopic m/z. Structural elucidation of THT GSH adduct product (RT: 52.3 – 54.5 min) in MS/MS spectrum using (C) CID, or (D) EAD fragmentation. Embedded subfigures in (D) are zoomed-in MS/MS spectra of (i) an individual GSH, (ii) THT + 1 Hydrolyzed Linker-PL product ion. The THT peptide has a full amino acid sequence of THTCPPCPAPELLGGPSVFLFPPKPK.

S6. Investigation of conjugation site-preference for forming disulfide bond with endogenous thiol-bearing molecules using CID fragmentation.

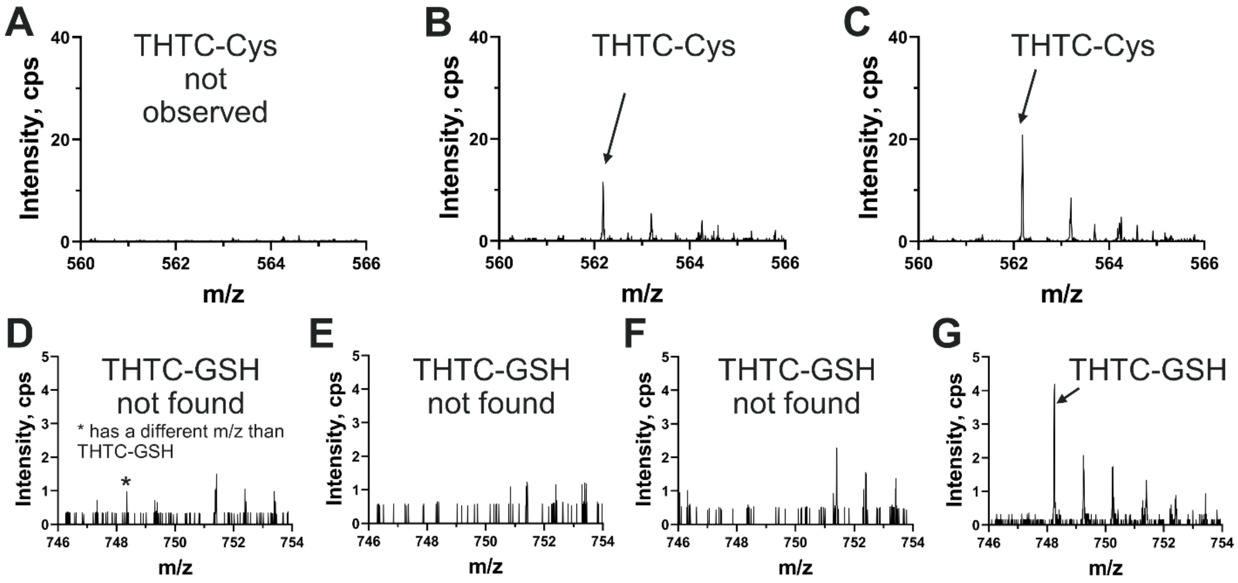

Figure S4. Investigation of conjugation site-preference for forming disulfide bond with endogenous thiol-bearing molecules using CID fragmentation. (A-C) MS/MS spectra of THT peaks 1-3 denoted in Figure 4, respectively. (D-G) MS/MS spectra of peaks 1-4 denoted in Figure S2, respectively.

S7. Characterization of the disulfide bond reformation between two Hc using both CID and EAD fragmentation

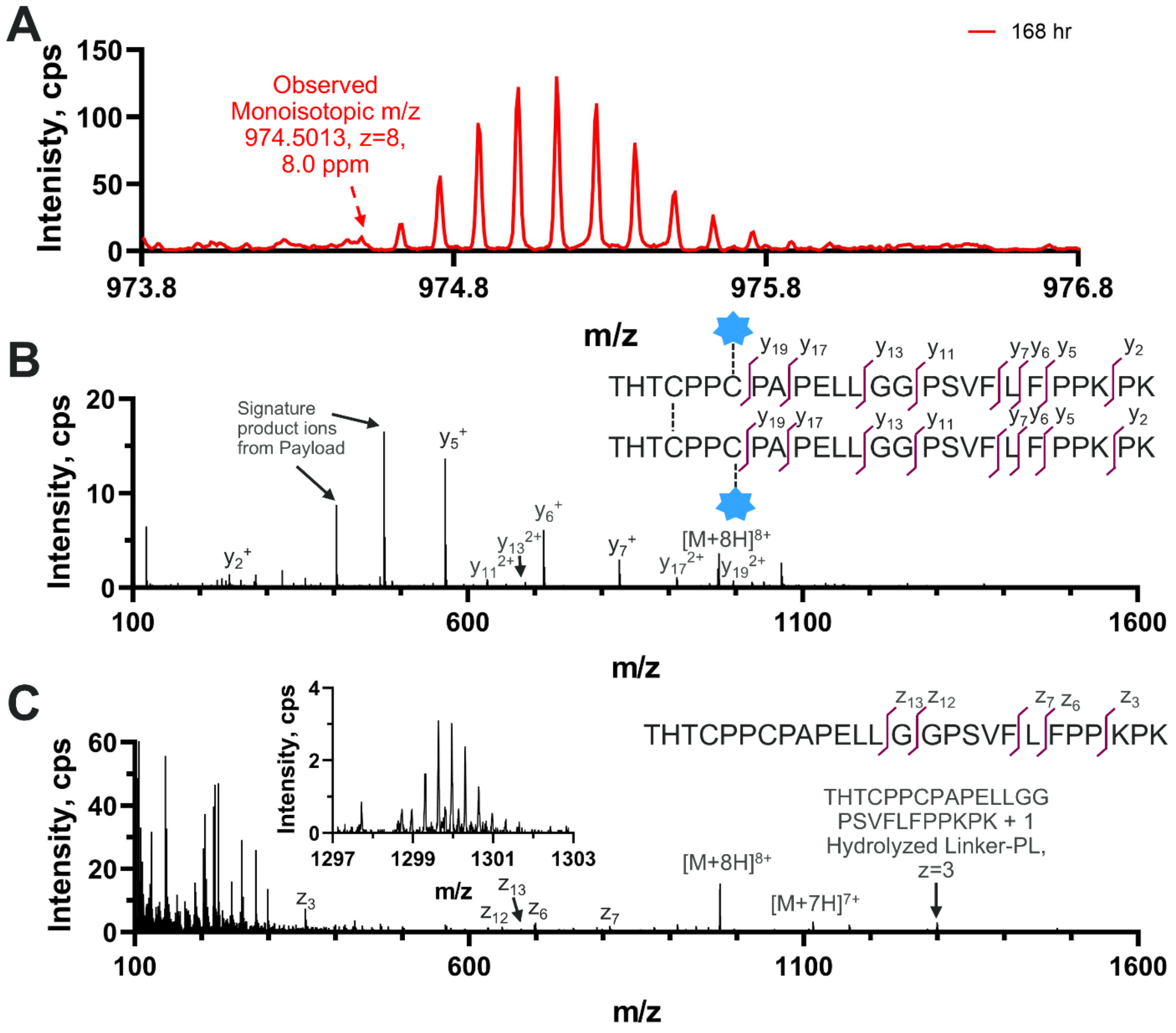

Figure S5. (A) MS spectrum of the biotransformation species with Hc + Hc disulfide bond reformed, represented as THT + THT structure. Its monoisotopic m/z may be obscured by the noise. MS/MS spectra of THT + THT using (B) CID, (C) EAD fragmentation. The embedded subfigure in (C) shows a zoomed-in MS/MS spectrum of THT +1 Hydrolyzed Linker-PL with z=3. It has a different charge state than the precursor ion. The y and z ions are labelled as in THT peptide.

S8. Structural confirmation of an intra-chain disulfide-linked Hc biotransformation species

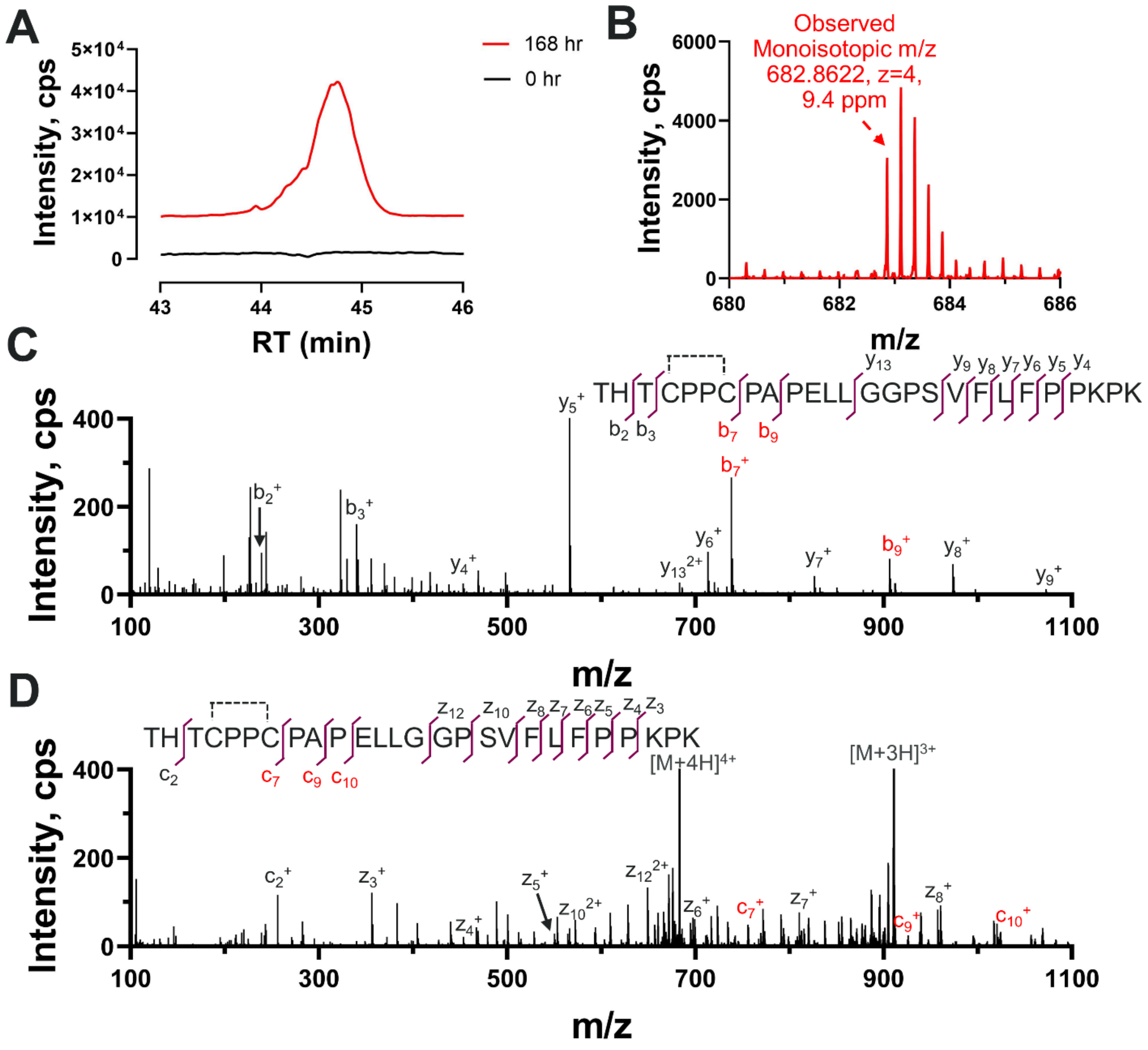

Figure S6. Structural confirmation of an intra-chain disulfide-linked Hc species (A) XICs of the intrachain disulfide-linked Hc species, represented as THT***C***PP***C***PAPELLGGPSVFLFPPKPK (two bolded C formed disulfide linkage). (B) MS spectrum of this biotransformed species. Dashed arrow points to its monoisotopic m/z. MS/MS spectra of scrambled disulfide-linked THT***C***PP***C***PAPELLGGPSVFLFPPKPK using (C) CID, (D) EAD fragmentation. The b/y and c/z ions are labelled as from THT peptide. The red color b and c ions show observed masses that are ~ 2 Da less than their theoretical masses, suggesting formation of disulfide bond.
